# Rpn (YhgA-Like) Proteins of Escherichia coli K-12 and Their Contribution to RecA-Independent Horizontal Transfer

**DOI:** 10.1128/JB.00787-16

**Published:** 2017-03-14

**Authors:** Anthony W. Kingston, Christine Ponkratz, Elisabeth A. Raleigh

**Affiliations:** New England BioLabs, Ipswich, Massachusetts, USA; University of Wisconsin—Madison

**Keywords:** DNA damage, conjugation, evolutionary biology, gene function, genomic instability, horizontal gene transfer, illegitimate recombination, protein function, protein motif, transposase

## Abstract

Bacteria use a variety of DNA-mobilizing enzymes to facilitate environmental niche adaptation via horizontal gene transfer. This has led to real-world problems, like the spread of antibiotic resistance, yet many mobilization proteins remain undefined. In the study described here, we investigated the uncharacterized family of YhgA-like transposase_31 (Pfam PF04754) proteins. Our primary focus was the genetic and biochemical properties of the five Escherichia coli K-12 members of this family, which we designate RpnA to RpnE, where Rpn represents recombination-promoting nuclease. We employed a conjugal system developed by our lab that demanded RecA-independent recombination following transfer of chromosomal DNA. Overexpression of RpnA (YhgA), RpnB (YfcI), RpnC (YadD), and RpnD (YjiP) increased RecA-independent recombination, reduced cell viability, and induced the expression of reporter of DNA damage. For the exemplar of the family, RpnA, mutational changes in proposed catalytic residues reduced or abolished all three phenotypes in concert. *In vitro*, RpnA displayed magnesium-dependent, calcium-stimulated DNA endonuclease activity with little, if any, sequence specificity and a preference for double-strand cleavage. We propose that Rpn/YhgA-like family nucleases can participate in gene acquisition processes.

**IMPORTANCE** Bacteria adapt to new environments by obtaining new genes from other bacteria. Here, we characterize a set of genes that can promote the acquisition process by a novel mechanism. Genome comparisons had suggested the horizontal spread of the genes for the YhgA-like family of proteins through bacteria. Although annotated as transposase_31, no member of the family has previously been characterized experimentally. We show that four Escherichia coli K-12 paralogs contribute to a novel RecA-independent recombination mechanism *in vivo*. For RpnA, we demonstrate *in vitro* action as a magnesium-dependent, calcium-stimulated nonspecific DNA endonuclease. The cleavage products are capable of providing priming sites for DNA polymerase, which can enable DNA joining by primer-template switching.

## INTRODUCTION

In prokaryotes, horizontal gene transfer (HGT; also called lateral gene transfer) is a massive force of evolutionary change and adaptation. It promotes the acquisition of new genes that allow bacteria to adapt to ecological niches and survive under stressful conditions when traditional gene regulation is not sufficient (1). To illustrate the magnitude of the issue, consider Escherichia coli. Approximately 40% of a typical E. coli genome and 90% of the E. coli species-wide pangenome consist of foreign gene islands (2–4), in the sense that they are not shared by all E. coli isolates. However, many aspects of HGT are still poorly understood, and its overall effect on genomic evolution is the subject of active research (5).

Both homologous and nonhomologous recombination processes contribute to pangenome assembly (6, 7). Homologous recombination is a universally conserved process mediated by strand transfer proteins, such as the RecA/RadA family of proteins. It acts efficiently to disseminate advantageous genetic material (7, 8), but its dependence on sequence identity (>96 to 97%) limits the exchange to close relatives (9). In contrast, nonhomologous recombination can come in many forms and is often less efficient but can operate across large phylogenetic distances because it does not depend on extensive DNA sequence similarity. The two processes complement each other: a nonhomologous recombination event can add a novel capability to one member of a population, and homologous exchange can then spread that capability within the population more efficiently than the original mechanism that introduced it (10).

Most nonhomologous gene addition mechanisms involve a DNA-mobilizing protein or complex that places its own gene(s) into a new location (5). Such action may also move cargo genes—nonmobile genetic material—along with the gene for the mobile element (11). Common examples include transposases (12), site-specific recombinases (13), and integrases (14).

The YhgA-like family (Pfam PF04754) (15) has been proposed to represent a class of DNA-mobilizing enzymes on the basis of bioinformatic analysis: the genes involved are sporadically distributed among a wide variety of bacteria, often with multiple paralogs in each genome (16). PF04754 is designated a putative transposase family (transposase_31) by the TigrFam (16) and Pfam (17) databases. Separately, members of this protein family were predicted to encode a PD-(D/E)XK phosphodiesterase domain (18, 19). This domain is prevalent in nucleases (20) but has also been found in enzymes connected to HGT (21, 22).

Though some YhgA-like proteins have been analyzed *in silico* (18, 19), we are the first to investigate these proteins experimentally. Our investigation centered on the five E. coli K-12 family members. These are renamed here to reflect their functional characterization presented below: RpnA (YhgA), RpnB (YfcI), RpnC, (YadD), RpnD (YjiP), and RpnE (YfaD). We show that overexpression of RpnA to RpnD increases recombination efficiency in our conjugal system, reduces cell viability in a *recA*-deficient background, and induces a reporter of DNA damage in a *recA* wild-type background, while RpnE is inactive in these assays. We then focus on the exemplar RpnA to show that the predicted PD-(D/E)XK domain is responsible for these phenotypes *in vivo* and that purified RpnA exhibits calcium-stimulated, magnesium-dependent DNA endonuclease activity *in vitro*. We also provide suggestive evidence that family member *rpnC* has been acquired twice at a syntenic location in enteric bacteria.

## RESULTS

### Background: the conjugal system and YhgA-like paralogs. (i) The conjugal system.

The Escherichia coli mating system consists of a tetracycline-resistant donor and a streptomycin-resistant recipient, both of which are RecA deficient. In the donor, an integrated F plasmid (Hfr) lacking vegetative replication functions promotes DNA transfer (see Fig. S1 in the supplemental material). Recombinants are selected with both drugs following the conjugal transfer of donor chromosomal DNA to the recipient. Recombinants result when a donor marker (*mrr*::*tetRA*) is added to or replaces a segment of the recipient chromosome. Under the basal condition with this system, most recombinants are found to be replacements (23). These recombination events occur at a low frequency (∼10^−10^/recipient/h), and the exchange can range from just a portion of the genome island originally interrogated (<16 kb) to over half of the genome (>2.4 Mb); a majority of events have replaced over 400 kb (23).

### (ii) YhgA-like proteins.

**FIG 1:**
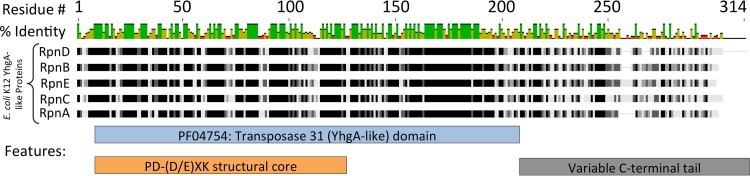
Alignment of the five E. coli K-12 YhgA-like proteins and their predicted domain structures. The RpnA (YhgA), RpnB (YfcI), RpnC (YadD), RpnD (YjiP), and RpnE (YfaD) sequences were derived from the E. coli K-12 genome and aligned. The chart on top gives the percent identity at each position of the alignment. Green, 100% identity; yellow, 30 to 70% identity; red, <30% identity. For each individual protein sequence, black bars denote 100% similarity to the consensus sequence, with progressively lighter bars indicating less similarity and gaps being represented as light gray lines. The location of the transposase_31 domain, as reported by the Pfam database (17), and the PD-(D/E)XK structural core identified by Knizewski et al. (19) are shown as boxes under the alignment.

The first experimental evidence linking YhgA-like proteins (PF04754) to HGT was discovered while testing this conjugal system. The PF04754 member *rpnD* (formerly *yjiP*) was investigated due to its close proximity to the genome island under study (24). Though *rpnD* did not appear to be a part of the island, its expression promoted the RecA-independent recombination encompassing it (23). The E. coli K-12 genome encodes 4 additional YhgA-like proteins (RpnA, RpnB, RpnC, and RpnE) with a conserved 5′ domain (Fig. 1).

### YhgA-like protein expression *in vivo* promotes RecA-independent recombination and damages the genome. (i) Increased RecA-independent recombination.

**FIG 2:**
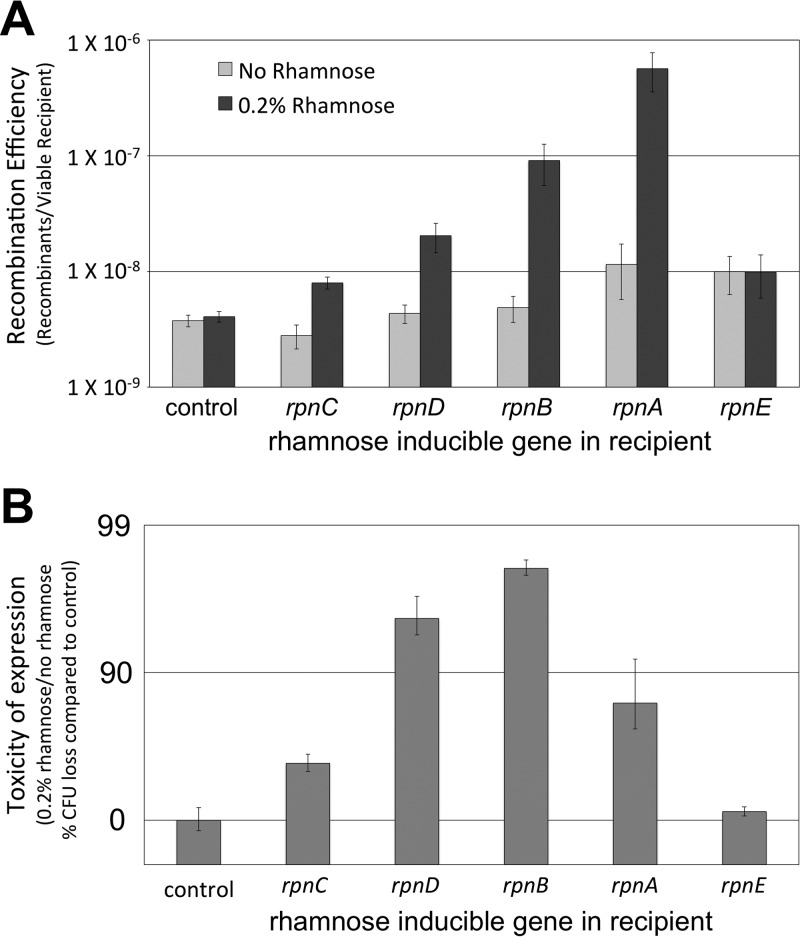
YhgA-like proteins increase recombination efficiency and are toxic. (A) Frequency of recombination during matings between the standard Δ*recA* donor (strain ER3435) and either the Δ*recA* recipient (control, ER3473) or a Δ*recA* recipient with a rhamnose-inducible overexpression construct (*rpnD*, ER3481; *rpnC*, ER3512; *rpnE*, ER3513; *rpnA*, ER3514; and *rpnB*, ER3511) that was uninduced (no rhamnose) or induced (0.2% rhamnose). (B) Toxicity of expression was measured as the percent reduction in the number of recipient CFU per milliliter during rhamnose treatment relative to that for the control during the matings indicated in panel A.

RecA-independent recombinants were recovered at higher rates upon overexpression of RpnA to RpnD but not RpnE. We fused each of the E. coli K-12 YhgA-like protein-encoding genes to the rhamnose-inducible promoter *rhaBp* and integrated these constructs into the Tn*7* attachment site of Δ*recA* recipient strains (23, 25). We mated these recipients to the Δ*recA* donor in media with and without 0.2% rhamnose and recorded the recombination efficiency as the proportion of recipients that acquired tetracycline resistance from the donor. Rhamnose had no significant effect on recombination efficiency in a control mating or when it induced the expression of *rpnE* (Fig. 2A). However, rhamnose-induced expression of *rpnC*, *rpnD*, *rpnB*, and *rpnA* significantly increased the recombination efficiency by 2.9-, 4.7-, 19-, and 49-fold, respectively (Fig. 2) (*P* = 0.002, 0.018, 0.037, and 0.029, respectively).

### (ii) Toxicity in Δ*recA* cells.

RpnA to RpnD were also toxic to the Δ*recA* recipients. After each mating, the number of CFU of the recipient per milliliter was counted to assess cell viability. The number of CFU per milliliter of the *rhaBp-rpnE* recipient resembled that of the control, but rhamnose reduced the number of CFU per milliliter of the *rhaBp-rpnC*, *rhaBp-rpnD*, *rhaBp-rpnB*, and *rhaBp-rpnA* recipients by 59%, 94%, 98.7%, and 98.0%, respectively (Fig. 2B). This cell toxicity could be related to the effect of YhgA-like protein expression on recombination efficiency, but the two factors were not completely correlated. For example, RpnA was the strongest activator of RecA-independent recombination but had the second weakest cell toxicity.

### (iii) Induced DNA damage (SOS) response.

YhgA-like proteins could be toxic to Δ*recA* cells due to DNA damage: the PD-(D/E)XK domain found in YhgA-like proteins is best known as the active site in restriction endonucleases (20), and RecA-deficient cells are hypersensitive to DNA damage.

Consistent with this hypothesis, all the active E. coli K-12 YhgA-like proteins induced a reporter of the SOS response when expressed in a Rec^+^ host. Plasmids with rhamnose-inducible constructs were introduced into an SOS indicator strain: *lacZ* was fused to the DNA damage-inducible *dinD* locus on the E. coli genome [*dinDp-lacZ*(Ts)] (26). All these strains except for the strain carrying *rhaBp-rpnE* gave a blue color on X-Gal (5-bromo-4-chloro-3-indolyl-β-d-galactopyranoside) plates when they were induced with rhamnose (data not shown).

**FIG 3:**
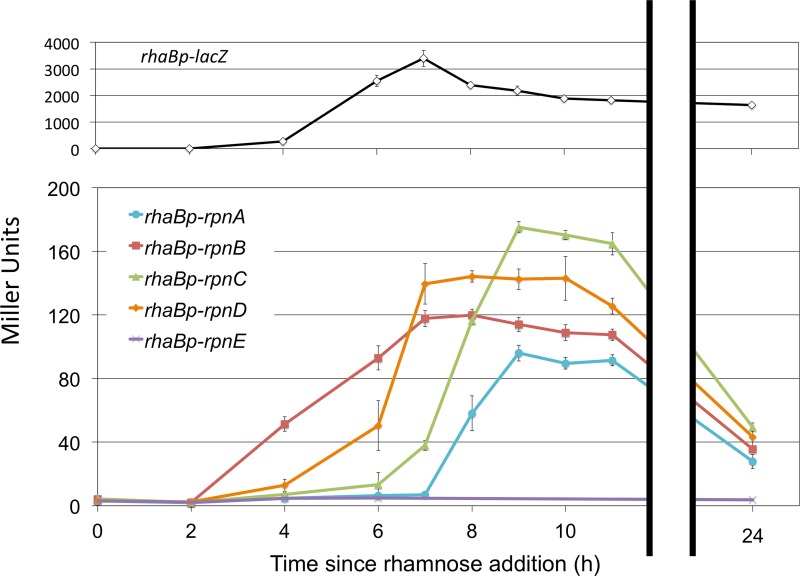
YhgA-like protein overexpression induces an SOS reporter in *rec*-positive E. coli. The expression magnitude and dynamics of the indirect induction of *dinD*::*lacZ* by Rpn proteins were compared with the direct induction of *rhaBp-lacZ* by rhamnose. Strains with inducible *rhaBp-rpnD* (ER3560), *rhaBp-rpnB* (ER3561), *rhaBp-rpnC* (ER3562), *rhaBp-rpnE* (ER3563), or *rhaBp-rpnA* (ER3564) and rhamnose-inducible *lacZ* (*rhaBp-lacZ*, ER3245) were grown in parallel and induced when the OD_600_ was 0.2. Aliquots were tested for β-galactosidase activity with time.

Quantitative β-galactosidase assays showed that the intensity of the SOS response induced by each YhgA-like protein (in Rec-positive [Rec^+^] strains) was proportionate to the cell-killing activity of the protein (in Rec-negative [Rec^−^] strains). To understand the induction kinetics and the dynamic range of this indirect assay, a rhamnose-inducible *lacZ* gene in the Rec^+^ strain was analyzed in the same way. The most lethal paralogs, RpnB and RpnD, were the first to activate the SOS-responsive *dinDp-lacZ*(Ts) reporter, at a time when the *rhaBp* promoter had just begun to activate *lacZ* (Fig. 3). RpnA and RpnC were substantially weaker in this regard, in that they activated the *dinDp-lacZ*(Ts) reporter only after the *lacZ* activity driven directly from the rhamnose promoter reached a maximum. Expression of *rpnE* for 24 h did not affect β-galactosidase activity, consistent with its lack of activity in other assays.

### (iv) Interpreting initial results.

Taken together, these findings establish that YhgA-like proteins participate in a RecA-independent recombination mechanism. The most direct evidence of such a mechanism is that YhgA-like protein expression increased productive recombination, while the cell toxicity and SOS induction phenotypes suggest that these proteins act on the recipient genome.

### Comparison of the YhgA-like family to known DNA damage processes using the conjugal system. (i) Comparison to the chain-terminating nucleotide AZT.

**FIG 4:**
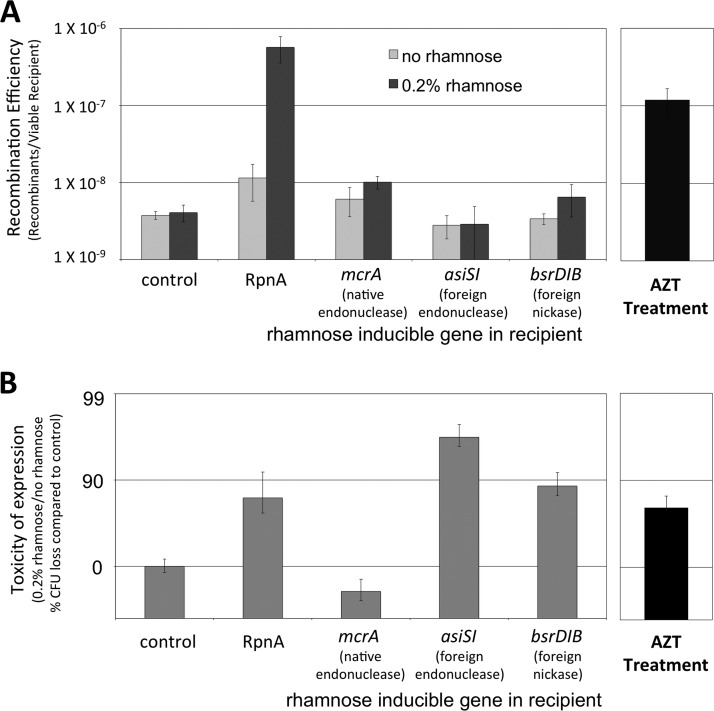
Site-specific endonucleases and AZT have disparate effects in this system. (A) Recombination efficiency: mating of the Δ*recA* donor (ER3435) with and without rhamnose induction of the Δ*recA* recipient (control, ER3473) or inducible *rpnA* (ER3514), *mcrA* (ER3533), *asiSI* (ER3535), or *bsrDIB* (ER3541) and with azidothymidine during mating (AZT; 2.5 ng/ml) (ER3435 × ER3473). (B) Toxicity of the expressed proteins/treatments during these matings.

Azidothymidine (AZT) is a thymidine analogue that acts as a chain terminator (27). It is known to promote template switching during replication (28). We earlier suggested the involvement of template switching to explain recombination in our system (23). Addition of AZT to a wild-type (WT) mating (ER3435 × ER3473) significantly increased the recombination efficiency: at a concentration high enough to reduce cell viability by 4.8-fold, the recombination frequency increased by 31-fold (*P* = 0.01) (Fig. 4).

### (ii) Comparison to nucleases.

Nuclease action alone is not sufficient to increase recombination efficiency in this system. We overexpressed the native endonuclease McrA, the foreign endonuclease AsiSI, and the foreign nickase BsrDIB in Δ*recA* recipients. McrA served as a negative control since the donor and recipient genomes are resistant to this endonuclease (29). As expected, it had no effect on recombination efficiency or cell toxicity when expressed (Fig. 4). Expression of AsiSI or BsrDIB reduced cell viability (by 97% and 88%, respectively), but recombination was not stimulated in either case.

### Differentiating basal recombination from RpnA-promoted recombination.

Our next goal was to determine whether YhgA-like proteins were increasing basal recombination events (those that already occur in the conjugal system) or were promoting a different pathway. Prior work had shown only that basal recombination events are infrequent and that recombinants carried replacements of large segments of genomic DNA (23).

**FIG 5:**
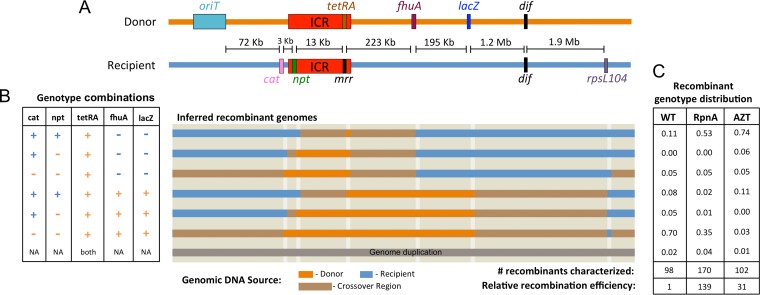
Distribution of genomic exchanges in recombinants. (A) Diagram of markers that distinguish the donor and recipient genomes and distances from the selected *tetRA* cassette. The chromosome segregation site *dif* is also shown. ICR, restriction enzyme gene cluster known as the immigration control region. (B) Recombinants were screened for the *cat*, *npt*, *mrr*, *fhuA*, and *lacZ* markers. Recombinants containing both *tetRA* and *mrr* were classified as having genome additions, and the results for these recombinants are not shown. Horizontal bars indicate the extent of donor DNA (orange) that we inferred replaced the recipient genome (blue) during the recombination event. NA, not available. (C) Proportion of recombinants in each class from a basal mating (WT; ER3435 × ER3473) with or without AZT treatment or from a mating in which *rpnA* was overexpressed (RpnA; ER3435 × ER3514). For the WT, most recombinants were created by large replacements of over 400 kb of genomic DNA. In the RpnA and AZT matings, large replacements were less frequent, and over half of the genomic replacements were within the 236-kb segment between the *npt* and *fhuA* markers flanking the selected marker, *tetRA*.

Here, we found a larger proportion of shorter (but still large) replacement segments when RpnA participates than when it does not. To estimate the size of replacement segments, we monitored markers located at various distances from the selected donor marker (*mrr*::*tetRA*) (Fig. 5A). The *npt* and *cat* resistance cassettes were within 16 kb proximal to the *mrr* locus in the recipient genome, and the *fhuA*::IS*2* and *lacZ*-positive (*lacZ*^*+*^) markers were 223 kb and 418 kb distal to the *mrr*::*tetRA* locus in the donor genome, respectively. We analyzed 96 and 170 recombinants produced under basal (WT) conditions and during RpnA (RpnA-promoted) overexpression, respectively, representing at least 9 independent matings in each case. The frequency of additions (those recombinants acquiring *tetRA* and also retaining *mrr* in a PCR screen) was under 4% for both conditions (Fig. 5B and C). Seventy percent of the WT recombinants received all the donor markers and therefore exchanged at least 434 kb of genomic DNA; only 11% received just the *tetRA* cassette (maximum exchange of 236 kb, less than 13 kb of which could be proximal to *tetRA*). In contrast, 35% of the RpnA-promoted recombinants received all the donor markers and 53% received just the *tetRA* cassette. The recombinant distribution with AZT treatment resembled that found with RpnA overproduction but was more pronounced; 74% of the recombinants acquired only the *tetRA* cassette.

Consistent with the changed recombinant distribution, deletion of the genes encoding the four active E. coli K-12 YhgA-like proteins in the recipient strain (producing Δ*rpnA*, Δ*rpnB*, Δ*rpnC*, and Δ*rpnD* strains) did not affect the frequency of recombinant formation (Fig. S2). These paralogs thus do not contribute to the basal recombination frequency or distribution. We also tested the potential role of the Rac prophage-encoded RecET recombinase. This is normally silent (30, 31), but rare unscheduled expression can be imagined. Deletion of the entire sequence for the prophage did not affect the frequency recombinant formation.

### Three segments of YhgA-like proteins determine *in vivo* activity.

YhgA-like proteins can be roughly divided into three segments: a predicted PD-(D/E)XK structural core (18, 19) which comprises the N-terminal portion of the conserved longer transposase_31 domain; the remaining portion of the transposase_31 domain, which is also highly conserved in the family; and a variable C-terminal tail (Fig. 1). We investigated the function of each segment by expressing a series of YhgA-like protein variants in the mating system.

### (i) The PD-(D/E)XK structural core.

**FIG 6:**
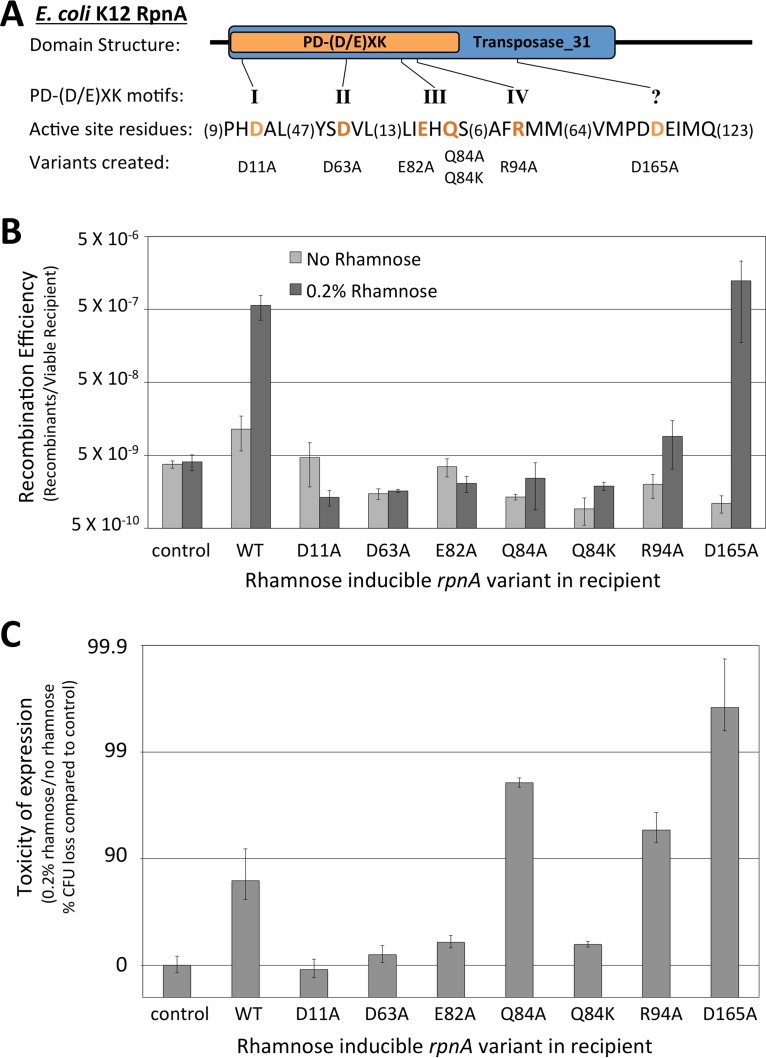
Phenotypes of mutated RpnA proteins *in vivo*. (A) The RpnA domain structure, PD-(D/E)XK core motifs, and active-site residues predicted by Knizewski et al. (19). RpnA variants with mutations in these active-site residues were created; an additional mutation in which a conserved PDDE motif was converted to PDAE (D165A) was made. (B) Recombination efficiency of matings between the Δ*recA* donor (ER3435) and either the control Δ*recA* recipient (ER3473) or Δ*recA* recipients with rhamnose-inducible *rpnA* or its mutants (WT, ER3514; D11A, ER3552; D63A, ER3553; E82A, ER3554; Q84A, ER3556; Q84K, ER3555; R94A, ER3557; and D165A, ER3558) with and without induction. (C) Toxicity of induction during these matings, reflected in viability decline.

The role PD-(D/E)XK structural core was probed by the use of mutations in predicted active-site residues. We mutated each predicted signature residue with alanine substitutions; in one case, we also changed a glutamine residue characteristic of this family but unusual in other PD-(D/E)XK enzymes (19) to lysine. We expected this mutant series (Fig. 6A) to exhibit low or diminished activity. The mutants were placed in the same expression environment as the parent and tested for the three phenotypes: increased recombination efficiency, cell toxicity, and SOS induction. The isolates carrying RpnA with the D11A mutation (RpnA-D11A), RpnA-D63A, RpnA-E82A, and RpnA-Q84K lost all three phenotypes (Fig. 6B and C). RpnA-Q84A and -R94A did not promote recombination but still reduced cell viability (by 98.1% and 94.6%, respectively, which made them 8.1- and 3.0-fold more toxic than WT RpnA, respectively). Both mutants elicited an SOS response on the plates as well (not shown).

### (ii) The non-PD-(D/E)XK portion of the transposase_31 domain.

The C-terminal 81 residues of the transposase_31 domain are not part of the PD-(D/E)XK structural core but are still highly conserved among YhgA-like proteins (Fig. 1) (17). In particular, a PDDEI motif within this segment is identical in all E. coli K-12 YhgA-like proteins (17). Mutating this acidic cluster to create RpnA-D165A unexpectedly yielded a hyperactive RpnA variant: it was 2.2-fold more effective than the WT in promoting recombination and 42-fold more toxic than the WT to cells (Fig. 6B and C). This high-activity mutation confirms the functional relevance of the non-PD-(D/E)XK portion of the transposase_31 domain.

### (iii) The variable C-terminal tail.

An RpnD variant lacking the last 45 residues of its variable C-terminal tail was substantially less active than WT RpnD but not completely dead. Expression of *rhaBp-rpnD* with a deletion of nucleotides 786 to 921 increased recombination (2.3-fold) and reduced cell viability (1.6-fold) compared with the results for the control strain (Fig. S3). These effects were statistically significant but lower than the effects of WT RpnD, which increased recombination 2.0-fold more (4.5-fold total) and was 7.8 times more toxic to cell viability.

### Migration history of YhgA-like proteins suggests independent *rpnC* (*yadD*) insertion events at the same place.

We undertook a limited reinvestigation of the distribution of *rpnC* (formerly *yadD*) to find support for its self-mobility. Our work flow is fully described in Text S1, and key findings are summarized here.

The distribution of *rpnC* orthologs is consistent with two separate introductions into the intergenic region between *panC* and *panD* (Text S1). We identified by BLAST analysis *panCD* DNA segments in Enterobacteriaceae with similarity to the sequence of the K-12 genome at the 5′ end of *panC* and the 3′ end of *panD*. A collection of 32 sequences, 18 of which had similarity to *rpnC*, were compared using nucleotide sequence alignment and phylogenetic tree construction (Fig. S4 and S5; Table S2). Two clusters were more divergent from each other than the flanking *panC* and *panD* homologs were. They could be visually distinguished by aligning the *panC-panD* intergenic regions and creating an evolutionary tree. Comparison of the phylogeny of these intergenic regions (Fig. S4B) with that of the flanking core genes (Fig. S4A) strongly suggested that two distinct versions of *rpnC* (*yadD*) were introduced separately into ancestral *pan* operons. Alternative explanations are described in Text S1.

### RpnA exhibits nonspecific DNA endonuclease activity *in vitro*. (i) Purification of RpnA.

RpnA and two RpnA variants were purified to characterize the activity of YhgA-like proteins *in vitro*. Purification by affinity and anion-exchange chromatography yielded chromatographically pure RpnA, RpnA-D63A (which is inactive *in vivo*), and RpnA-D165A (which is hyperactive *in vivo*).

### (ii) RpnA has low DNA endonuclease activity.

**FIG 7:**
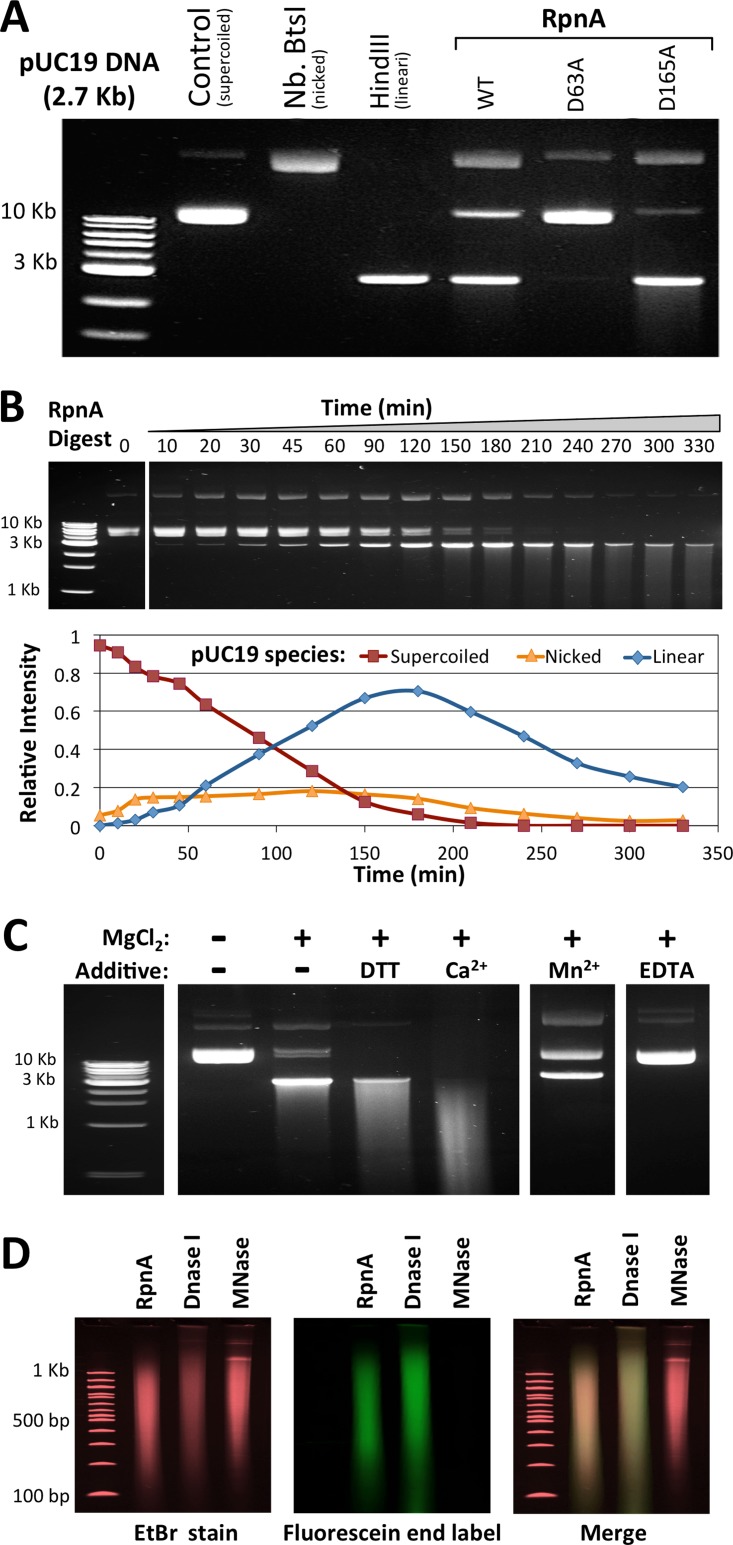
*In vitro* analysis of RpnA endonuclease activity. (A) WT RpnA cleaves pUC19, RpnA-D63A does not cleave pUC19, and RpnA-D165A is more active on pUC19. The pUC19 DNA (29 nM, 50 μg/ml) is initially supercoiled but can be relaxed by nicks, linearized by double-strand cleavage, or cleaved further. The supercoiled (control), relaxed (Nb.BtsI), and linear (HindIII) forms are indicated. pUC19 was treated with RpnA-inactive RpnA-D63A or hyperactive RpnA-D165A (15 μM, 45 min). (B) Time course of an RpnA (7.5 μM)-pUC19 (29 nM) digest. Band intensity was compared to determine the relative amounts of supercoiled, nicked, and linear pUC19 at each time point. Over 90% of the supercoiled pUC19 was digested within 180 min. (C) RpnA endonuclease activity depends on divalent cation and is stimulated by Ca^2+^. The reaction buffer was 50 mM NaCl and 10 mM Tris, pH 8.0; the indicated additives were at 10 mM each. RpnA at 3.8 μM was added for 18 h. (D) RpnA cleavage products provide a DNA polymerase primer. pUC19 was digested with RpnA, DNase I, or micrococcal nuclease (MNase) to produce similar smears and then incubated with fluorescein-labeled dNTPs and the Klenow fragment of DNA polymerase. DNA was visualized by ethidium bromide (EtBr; left) or fluorescein (middle), with the two signals being merged at the right. RpnA- and DNase I-digested DNAs were effectively labeled, but micrococcal nuclease-digested DNA was not.

Purified RpnA exhibited low but detectable DNA endonuclease activity *in vitro*. Supercoiled pUC19 was initially used as the DNA substrate because a single nick relaxes the plasmid, a single cleavage linearizes it, and all three species (supercoiled, relaxed, and linear) can be easily distinguished on an agarose gel (Fig. 7A). RpnA initially digests pUC19 to the linear species with a small increase in the amount of nicked plasmid. Time course assays showed that less than 20% of the supercoiled plasmid is nicked at any one time (Fig. 7B), and the entire plasmid is eventually digested to a smear of DNA. In contrast, the well-characterized and highly active nonspecific nicking enzyme DNase I (32) converted 40% of the total DNA to the nicked product before substantial linearization occurred and digested DNA 2.8 × 10^7^-fold faster than RpnA (Fig. S6A).

### (iii) Mutated RpnA proteins display activity consistent with their *in vivo* phenotypes.

With pUC19 and the linear bacteriophage λ DNA substrate, RpnA-D63A had negligible activity (<0.006-fold the WT activity) and RpnA-D165A digested the substrates ∼2-fold faster than the WT enzyme (Fig. 7A and S6B to D). Since the activities of these variants mirror the effects of their overexpression *in vivo*, we conclude that the purification strategy successfully removed potentially contaminating nucleases and that these assays reflect the RpnA endonuclease activity.

### (iv) Digestion patterns do not suggest sequence specificity.

No evidence of sequence specificity was detected with the substrates used, in that no banding pattern was detected (Fig. 7 and S6). In long digests, RpnA degraded DNA substrates to small fragments: bacteriophage λ DNA was reduced to a smear of between 100 and 500 bp on an agarose gel (Fig. S7A), which disappeared when four times as much RpnA was used. We did not observe a band of recalcitrant DNA that would constitute a sequence resistant to RpnA digestion in these extended digests. Similar results were obtained when RpnA was incubated with E. coli K-12 genomic DNA or a 2-log DNA ladder (Fig. S7B and C). The disappearance of the upper bands of the DNA ladder first (Fig. S7C) is consistent with primary endonuclease action; exonuclease action would affect small bands first.

### (v) DNA conformation does not affect RpnA activity; dsRNA is not cleaved.

Substrates sensitive to RpnA included covalently closed circular pUC19, linear molecular weight ladders, full-length bacteriophage λ DNA, and single-stranded DNA (ssDNA) of the M13 virion. When ssDNA and double-stranded DNA (dsDNA) substrates were digested together or separately, the two substrates were digested at comparable rates (Fig. S7D and E). RpnA did not exhibit a preference for nicked pUC19 or for the cruciform extrusion in pUC(AT) (data not shown). No digestion was observed with a double-stranded RNA (dsRNA) ladder after 18 h (Fig. S7F). In our hands, single-stranded RNA was too unstable to be assessed in this way (not shown).

### (vi) RpnA is a broadly active Mg^2+^-dependent enzyme with unusual stimulation by Ca^2+^.

Buffer optimization yielded the RpnA buffer described in Materials and Methods. The full process by which we developed this buffer is described in Fig. S8, and key findings derived from this process are highlighted below.

As is true with most nucleases (33), Mg^2+^ was required: removal of MgCl_2_ by omission or by adding EDTA inactivated the enzyme (Fig. 7C). Mn^2+^ supported some action of RpnA in the absence of Mg^2+^ (Fig. S8C) but was inhibitory in its presence, as was Zn^2+^ (Fig. S8B). RpnA activity was slightly higher in the presence of dithiothreitol (DTT) and β-mercaptoethanol, as might be predicted from the cysteine content (5 residues; Fig. 7B and S8B).

Surprisingly, Ca^2+^ stimulated activity 6-fold (Fig. 7B and S8D). This was unusual because Ca^2+^ usually inhibits PD-(D/E)XK nucleases by competing with Mg^2+^ for the active site (33). With bacteriophage λ DNA as the substrate, RpnA activity was the highest when the Ca^2+^ concentration] was 1 to 2 times the Mg^2+^ concentration, but at higher ratios calcium was inhibitory (Fig. S8E).

RpnA tolerates a wide range of pH and salt. Using bacteriophage λ DNA as a substrate, we found activity between pH 7.5 and pH 10.5, with maximal activity occurring at a pH of 9.0 (Fig. S8F). NaCl concentrations ranging from 0 to 200 mM were acceptable, with a broad plateau taking place between 10 mM and 75 mM (Fig. S8G).

### (vii) RpnA cleavage results in 3′ hydroxyl DNA ends.

Fluorescent end-labeling experiments revealed that RpnA cleavage produces fragments extendable by DNA polymerase. pUC19 was digested to a smear of DNA ranging from 100 bp to 1 kb with either RpnA, DNase I, or micrococcal nuclease. Cleavage products were then incubated with the Klenow fragment and fluorescein-labeled deoxynucleoside triphosphates (dNTPs). The positive-control DNase I-digested smear had a strong fluorescent signal, confirming that the polymerase could effectively label 3′ hydroxyl ends, while the 3′ phosphate ends of the negative-control micrococcal nuclease-digested DNA (34) were not labeled at all (Fig. 7D). The RpnA-digested DNA exhibited a fluorescent signal, confirming the presence of 3′ hydroxyl ends.

## DISCUSSION

### Overproduction phenotypes implicate YhgA-like proteins in DNA transactions.

When they were artificially overproduced, four of the five endogenous E. coli K-12 paralogs in the YhgA-like transposase_31 family promoted RecA-independent recombination (Fig. 2A) and produced DNA damage *in vivo* in RecA^+^ cells (Fig. 3), likely accounting for their toxicity in RecA^−^ cells (Fig. 2B). These proteins were not responsible for the basal level of RecA-independent recombination, since the multiple-deletion strain lacking the four active paralogs showed the same level of recombinant formation as the wild type (see Fig. S2 in the supplemental material). The prophage-encoded *recET* recombination system was also not required for basal recombinant formation (Fig. S2).

Recombinants produced under both basal and RpnA-stimulated conditions carried large segmental replacements of recipient genes with donor genes, rather than additions of donor DNA to the recipient genome. Those formed with overproduced RpnA were smaller, on average, than those formed in its absence (Fig. 5). This distribution agrees with that observed with the overproduced paralog RpnD (23). The change in distribution strongly suggests that these proteins play a role in generating both proximal and distal crossover events, when present.

### *In vitro* analysis of *RpnA* reveals a novel nuclease activity.

The transposase annotation of YhgA-like proteins implies a transesterase activity able to reconnect the phosphodiester bonds in donor and recipient strands, sometimes via a protein-DNA covalent intermediate (12–14). However, nuclease activity was also expected from bioinformatic assignment of this family to the PD-(D/E)XK nuclease clan (Fig. 1). This clan includes structure-specific nucleases, exonucleases, and auxiliary transposon components as well as large numbers of restriction endonucleases. One homing endonuclease belongs to this clan, although most such mobile, highly specific nucleases belong to other nuclease clans (14, 35).

RpnA exhibited an *in vitro* nuclease activity plausibly related to the biological properties described above (Fig. 7). A mutation abrogating the recombination, toxicity, and DNA damage phenotypes (Fig. 6) also destroyed DNA degradation *in vitro*, while a different mutation that enhances those phenotypes also enhanced DNA degradation (Fig. 7A and S6B to D). RpnA had low nuclease activity compared to that of the digestive enzyme DNase I and did not show evidence of sequence specificity in the degradation process. Both dsDNA and ssDNA were substrates (Fig. S7D and E). The primary action could be either nicking or double-strand cleavage: some members of the PD-(D/E)XK clan nick two strands sequentially (36, 37), others cut two strands in concert (37), and others nick in collaboration with partner proteins (22, 38) or act at structural features (39, 40).

The fact that RpnA action provided a 3′ hydroxyl (Fig. 7D) was expected from the general properties of the PD-(D/E)XK family of enzymes (41–43) and provides further support for the assignment of PF04754 to this clan. It is also consistent with recombination models that rely on polymerase template switching at the cleavage site, since the 3′ end provides a polymerase-priming capacity without further processing.

RpnA is novel in its stimulation by Ca^2+^ (Fig. 7C and S8E). We could find no report of a PD-(D/E)XK nuclease stimulated by Ca^2+^ or any other intracellular Ca^2+^-stimulated bacterial nuclease at all. This result strongly implies two metal-binding sites in the protein, which has plenty of precedent in general, but reports are in conflict for PD-(D/E)XK nucleases (35, 41).

Though several types of Ca^2+^-stimulated/dependent nucleases have been discovered in eukaryotes (32, 44), prokaryotic examples are rare. PD-(D/E)XK enzymes are usually inhibited by Ca^2+^ because it competes with an essential Mg^2+^ ion at the active site (33), as it indeed did for RpnA when it was present in sufficient excess (Fig. S8E). The only Ca^2+^-dependent bacterial nuclease family that we found in the literature is the secreted micrococcal nuclease family (also known as Staphylococcus nuclease [SNase; PF00565]) (17) or related enzymes with an SNase-like domain (IPR016071) (45). These are quite distinct from RpnA: they require Ca^2+^ for activity, while RpnA is only stimulated by it; the SNase fold responsible for nuclease activity is structurally distinct from the PD-(D/E)XK domain; and most importantly, the products of SNase cleavage carry 3′ phosphate ends (34), while RpnA cleavage yielded 3′ hydroxyl ends that allowed DNA polymerase action (Fig. 7D).

A demonstration of calcium regulation *in vivo* is lacking at present, yet the effect of calcium on RpnA could be relevant to its role in recombination. Most studies estimate the intracellular calcium concentration in prokaryotes to be from 200 to 300 nM (46, 47), which is much lower than both the millimolar concentrations of Ca^2+^ required to activate RpnA *in vitro* (Fig. S8E) and the concentration of cytosolic Mg^2+^ (33). However, studies have shown that E. coli can raise total Ca^2+^ levels (48) and may be able to direct Ca^2+^ to specific regions of the cell (46). It is therefore possible that E. coli directs Ca^2+^ to RpnA to promote DNA transactions.

### Interpreting recombinant formation in the conjugal system.

The recombination events observed here may require collaboration with other endogenous DNA transaction proteins. The recombination transaction is not set in train simply by introducing DNA cleavage: damaging the recipient genome with a restriction endonuclease or a nicking enzyme did not increase recombinant formation (Fig. 4). Similarly, the degree of damage reflected in toxicity did not directly correlate with effective recombination, whether comparing paralogs (Fig. 2 and 3) or comparing mutated enzymes (RpnA-Q84A and -R94A) (Fig. 6).

YhgA-like proteins could nevertheless be causing DNA damage that provokes further processing. This damage could simply be the continued association of the nuclease with its site of action, resulting in a polymerase roadblock. Candidates for repair processing include primase (*priA*), involved in replisome assembly at stalled forks (49); DnaK, required for RecA-independent replication fork repair (50); or YoaA, a helicase thought to assist with the removal of blocked termini by displacement of the primer terminus (51). Single-strand annealing processes to promote primer-template switching (as observed in Salmonella [52]) and inducible RecA-independent repair mechanisms (such as the RpoS-mediated response [53]) could also participate in recombinant formation.

### AZT-stimulated recombination: support for a template-switch model of RpnA-stimulated recombination.

One model for recombination involves polymerase template switching, in which a 3′ end is freed from one homolog, anneals to the other homolog, and is extended by DNA polymerase. At the proximal crossover, such an event would connect the donor's antibiotic resistance cassette to the recipient replication origin. A similar event distal to the donor antibiotic resistance marker would complete the substitution. RpnA could be contributing to these switching events through genomic disruptions that lead to polymerase dissociation or by the creation of free 3′ ends that dissociated polymerase can act on.

The effect of azidothymidine (AZT) on the conjugal system is compatible with this model. AZT is a chain-terminating thymidine analog that causes DNA polymerase to dissociate from the genome and reveals ssDNA gaps to which the polymerase primer can anneal (54). This activity increases the frequency of template-switch-generated mutations in E. coli, which are conceptually similar to the template-switching events proposed by our model (28). Switching provoked by AZT would therefore be expected to increase the frequency of RecA-independent recombination and, if it is frequent enough, could reduce the overall size of genomic exchanges. Both AZT treatment and RpnA expression increased the recombination frequency (Fig. 2 and 4) and reduced the size of genomic exchange (Fig. 5). In this respect, the model is supported.

### Searching for a true target site: site specificity of gene location for an *rpnA* paralog.

Although RpnA degrades DNA with low activity and little to no specificity with the limited sequence universe tested *in vitro*, YhgA-like proteins might target specific DNA sites *in vivo*, as do homing endonucleases (35). Unlike insertion sequence (IS) elements, the genes for members of the RpnA family do not often move within laboratory lineages, making it problematic to identify sites of action. However, genome mining can suggest possibilities. Bioinformatic analysis of the *rpnC* (*yadD*) gene distribution suggests two independent insertions into the same genomic locus among the Enterobacteriaceae (Fig. S4). Acquisition could involve either the autonomous action of the enzyme to move its own gene or an action to import a copy from a distant relative by stimulating localized recombination or mutagenesis. The variable C-terminal tail of YhgA-like proteins (Fig. 1) might determine different DNA sequence preferences, resulting in different insertion positions for the different paralogs. Variable domains often function in sequence recognition (55, 56). Our own experimentation has shown for RpnD that truncation of the tail reduces but does not eliminate the recombination efficiency and cell toxicity *in vivo* (Fig. S3).

### Assignment of function to the uncharacterized PF04754 protein family.

The results of our genetic and biochemical studies support the assignment of a nuclease function to this family, broadly validating the predicted motif. The low activity and lack of sequence specificity among the tested paralogs are consistent with the possibility that these enzymes are eroded versions of ancient mobile elements. Limited reinvestigation of the phylogeny of *rpnC* (*yadD*) in its gene neighborhood is compatible with independent insertion at the same position on two occasions, leaving open the possible sequence specificity of the primary action, followed by the loss of activity due to the absence of selection.

The properties reported here could then represent secondary phenotypes, similar to the off-target effects of a homing endonuclease (35, 57) or transposase (e.g., see reference 58). Given the ubiquity of the family, such off-target effects may be relevant to genome island assembly, generating substrates for microhomology-mediated sequence assembly.

### Conclusion: the YhgA-like family represents a novel class of DNA-active proteins.

The experimental data presented in this paper confirm the biological relevance of the YhgA-like family to gene mobility. The fact that YhgA-like protein expression increases RecA-independent recombination is the central piece of evidence supporting this argument because it directly shows these proteins contributing to HGT events. The results of the cell toxicity, SOS induction, and *in vitro* nuclease activity experiments further confirm that YhgA-like proteins interact with DNA and suggest an HGT mechanism that involves DNA cleavage to create polymerase-extendable ends. A distinguishing property is that RpnA-promoted recombinants tend to exhibit exchanges of DNA shorter than those that occur with naturally occurring *recA*-independent recombination in the system.

## MATERIALS AND METHODS

### Strains, plasmids, and growth conditions.

All strains, plasmids, and oligonucleotides used in this study are listed in Table S1 in the supplemental material. Bacteria were routinely grown in liquid Luria broth (LB; 10 g/liter tryptone, 5 g/liter yeast extract, 10 g/liter NaCl, 1 g/liter dextrose, 1 g/liter MgCl_2_·6H_2_O) or rich broth (RB; 10 g/liter tryptone, 5 g/liter yeast extract, 5 g/liter NaCl, pH 7.2) medium at 37°C with vigorous shaking or on solid LB or RB medium containing 1.5% agar with appropriate selection. Ampicillin (Ap; 100 μg/ml), streptomycin (Sm; 100 μg/ml), kanamycin (Kn; 40 μg/ml), chloramphenicol (Cm; 30 μg/ml), and tetracycline (Tc; 20 μg/ml) were used for selections and screens. Where appropriate, 40 μg/μl X-Gal (5-bromo-4-chloro-3-indolyl-β-d-galactopyranoside) was added to score the *lac* phenotype. Plasmids were prepared from E. coli Turbo cells (catalog number C2984; New England BioLabs [NEB]). For temperature-sensitive plasmids, incubation at 30°C was used to permit plasmid replication and incubation at 42°C was used to remove the plasmid. Integrated pDEL-R6K vectors (59) were excised by plating on LB supplemented with 50 g/liter sucrose, 0.5 mM IPTG (isopropyl-β-d-thiogalactopyranoside), and 0.2% rhamnose.

### Genetic and molecular techniques.

Linear and plasmid DNA transformations were performed as described previously (60), as were P1*vir* transductions (61). DNA constructs were created using an NEBuilder HiFi DNA assembly kit (catalog number E5520; NEB). Chromosomal gene deletions were generated using a bacteriophage λ Red recombinase system (62) or a Fast genome engineering system (59). All PCR products used in the constructions were generated using E. coli MG1655 chromosomal DNA as the template, and the sequences of all strains were verified by PCR and/or sequence analysis (NEB DNA Sequencing Facility). PCRs used to generate sequencing templates or genetic constructions were performed with Q5 high-fidelity DNA polymerase (catalog number M0491; NEB), while diagnostic PCRs used the Hot Start *Taq* 2× master mix (catalog number M0496; NEB).

### Matings.

Donor and recipient cultures were grown at 37°C to an optical density at 600 nm (OD_600_) of ∼1.0 in RB with shaking. Mating was initiated by mixing the cultures in a 1:1 ratio and lasted for 18 h at 37°C. For rhamnose induction, 0.2% l-rhamnose monohydrate (catalog number R3875; Sigma-Aldrich) was added to the mating mixture at the start of mating. For AZT treatment, 2.5 ng/ml 3′-azido-3′-deoxythymidine (catalog number A2169; Sigma-Aldrich) was added to the mating mixture at the start of mating.

Mating mixtures for donors (Tc), for recipients (Sm), and for recombinants (Tc and Sm) were plated at dilutions that would yield at least 10 to 100 colonies per plate. In matings with a low recombination efficiency, up to 6 ml of the mating culture was centrifuged, resuspended in residual broth, and spread on multiple recombinant selective plates. Plates were incubated for 48 h at 37°C, and the colonies were counted to calculate the recombination efficiency and cell survival. Unmated donor and recipient cultures were subjected to identical procedures to determine the number of CFU per milliliter in unmated cells.

### Characterization of recombinants.

Recombinant colonies were purified once on the same selection medium and tested for markers that distinguish the donor and recipient genomes.

### *lacZ* screen for large replacements.

To score the frequency of large replacement recombinants among the total, we tested for the presence of *lacZ*, located 418 kb distal to the selected *tetRA* cassette in the donor genome but absent in the recipient (Fig. S1). RB X-Gal plates were incubated at 37°C for 2 days; blue colonies were classified as *lacZ*^+^.

### Antibiotic screens.

All recombinants were screened for unselected drug markers chloramphenicol (*cat*) and kanamycin (*npt*).

### PCR screens.

The *mrr* locus was screened with primers pER91 and pER92; the *fhuA* locus was screened with primers oTK148 and oTK149.

### Assessing the SOS response.

We tested the effect of YhgA-like protein overexpression on the SOS response using the *dinD-lacZ* DNA damage response reporter (63, 64). The *dinD2*::MudI1734 [Kan^r^ *lacZ*(Ts)] version of this reporter isolated by Piekarowicz et al. (65) was used because the indication is less ambiguous than that obtained with the leaky wild-type *dinD-lacZ* reporter (66). RB X-Gal plates with or without 0.2% rhamnose were used for initial assessment. Quantitative assays used cultures grown to an OD_600_ of 0.2 in RB at 30°C. Rhamnose (0.2%) was added, and growth was continued at 30°C with continuous shaking. Samples collected at various times after rhamnose addition were lysed by sonication, and β-galactosidase assays were performed (67).

### Characterizing RpnA *in vitro*. (i) Purifying RpnA and its variants.

The expression and purification of RpnA employed the Impact system (catalog number E6901; NEB) (68). Briefly, *rpnA* was inserted into the pTXB1 vector (pTK038) and transformed into the E. coli T7 expression strain (strain ER2566; NEB). RpnA was purified from the resulting strain (ER3573) as described in the kit's manual and then concentrated with a Vivaspin20 centrifugal ultrafiltration device (molecular weight cutoff, 10,000; GE Healthcare Life Sciences), dialyzed overnight in diluent A (catalog number B8001S; NEB), and kept at −20°C.

RpnA purified with the Impact system was further purified by anion-exchange chromatography. Concentrated protein was diluted to 50 ml in buffer (20 mM Tris-HCl, pH 8.0, 100 mM NaCl) and loaded onto a HiTrap Q HP 5-ml column using an Äkta fast-performance liquid chromatography system (model P-920; GE Healthcare Life Sciences). The protein was eluted with a 150-ml linear gradient of 100 to 600 mM NaCl over 38 fractions. The majority of the sample eluted as a single broad peak, which was pooled, concentrated, and dialyzed in diluent A to a final concentration of ∼10 mg/ml.

The final purified product was confirmed to be RpnA via liquid chromatography (LC)-mass spectrometry (MS). The single band of the protein sample isolated by SDS-PAGE was excised, digested with trypsin, and injected onto a 25-cm 3-μm C_18_ analytical column with a 1/4-cm Poros R1 plug. LC was accomplished with a Proxeon Easy-nLC II liquid chromatograph (Thermo Fisher), and MS data were collected from an LTQ Orbitrap XL mass spectrometer (Thermo Fisher) using a 60-min collision-induced dissociation/electron transfer dissociation (CID/ETD) data-dependent method. Data were analyzed with Proteome Discover (version 2.0) software (Thermo Fisher) and searched against the E. coli proteome to identify RpnA as the major protein in the sample.

Purification of the RpnA variants followed a similar protocol, in which expression strains ER3609 (RpnA-D63A) and ER3610 (RpnA-D165A) were used. The purities of the final products were confirmed by SDS-PAGE.

### (ii) Assessing RpnA activity.

RpnA endonuclease activity was determined by measuring DNA digestion with Tris-borate-EDTA (TBE)–agarose (1.5% or 0.75%) visualization by UV with ethidium bromide (EtBr) staining. Unless otherwise noted, the reaction conditions were as follows: purified protein and DNA substrate were combined in standard RpnA buffer (10 mM Tris-HCl, pH 9.0, 50 mM NaCl, 10 mM MgCl_2_, 15 mM CaCl_2_, 1 mM DTT) and incubated at 37°C. Substrates (all from NEB) were pUC19 (catalog number N3041), bacteriophage λ DNA (catalog number N3011), M13mp18 ssDNA (catalog number N4040), a dsRNA ladder (catalog number N0363), pUC19 nicked with Nb.BtsI (catalog number R0707), or pUC(AT), a pUC derivative carrying an extruded cruciform (69). Reactions were stopped with heat (70°C 10 min) or EDTA (30 mM). The 1.5% gels were run at 150 V for pUC19; the 0.75% gels were run at 100 V for bacteriophage λ DNA. Densitometry analysis with ImageJ software was used to compare the intensities of the DNA bands. Time course assays were used to determine relative activities by comparing the time needed to linearize 90% of pUC19.

### (iii) Labeling of digested DNA with fluorescein-labeled dNTPs.

pUC19 was digested to a smear of DNA ranging from 100 to 1,000 bp using either RpnA, DNase I (catalog number M0303S; NEB), or micrococcal nuclease (catalog number M0247; NEB). DNA smears (1 μg) were incubated with dTTP, dATP, fluorescein–12-dCTP (catalog number NEL434001EA; PerkinElmer), fluorescein–12-dGTP (catalog number NEL496001EA; PerkinElmer), and the E. coli polymerase I Klenow fragment (catalog number M0210S; NEB) at 37°C for 30 min. Purified DNA was run on a 6% TBE-polyacrylamide gel; labeling was visualized using a Typhoon 9400 laser scanner (excitation wavelength, 488 nm; emission wavelength, 520 nm; GE Healthcare Life Sciences). Total DNA was visualized by UV after EtBr staining.

### Statistical analysis.

All genetic experiments were performed with a minimum of three biological replicates. Unless otherwise noted, data are presented as the mean ± standard error. Unpaired Student's *t* tests were used for statistical evaluation. A *P* value of ≤0.05 was considered statistically significant.

## Supplementary Material

Supplemental material
