# Supplementary material for: Rpn (YhgA-Like) Proteins of Escherichia coli K-12 and Their Contribution to RecA-Independent Horizontal Transfer
Source: J Bacteriol. 2017 Mar 14;199(7):e00787-16. doi: 10.1128/JB.00787-16 (PMC5350276; doi:10.1128/JB.00787-16)
Supplement: Supplemental material [file supp_199_7_e00787-16__index.html]

Supplemental material 

# Rpn (YhgA-Like) Proteins of Escherichia coli K-12 and Their Contribution to RecA-Independent Horizontal Transfer

## Supplemental material

- Supplemental file 1 -

  Fig. S1 (Model system for studying RecA-independent recombination events), S2 (Effect of removal of *rpn* genes or silent recombination genes of Rac on basal recombination), S3 (RpnD C-terminal tail removal and protein activity), S4 (Phylogenetic context of the conserved *panC-panD* CDS and intergenic region), S5 (Mauve aligment of *panCD* intergenic regions), S6 (Kinetics of DNase I and RpnA variants), S7 (RpnA nonspecific DNA endonuclease), and S8 (RpnA endonuclease buffer optimization), Text S1, and Tables S1 (Strains, plasmids, and oligonucleotides) and S2 (*Enterobacteriaceae panCD* segments analyzed to investigate *rpnC*/*yadD* distribution)

  PDF, 2.1M
