# Supplementary material for: Rpn (YhgA-Like) Proteins of Escherichia coli K-12 and Their Contribution to RecA-Independent Horizontal Transfer
Source: J Bacteriol. 2017 Mar 14;199(7):e00787-16. doi: 10.1128/JB.00787-16 (PMC5350276; doi:10.1128/JB.00787-16)
Supplement: Supplemental material [file JB.00787-16_zjb999094343s1.pdf]

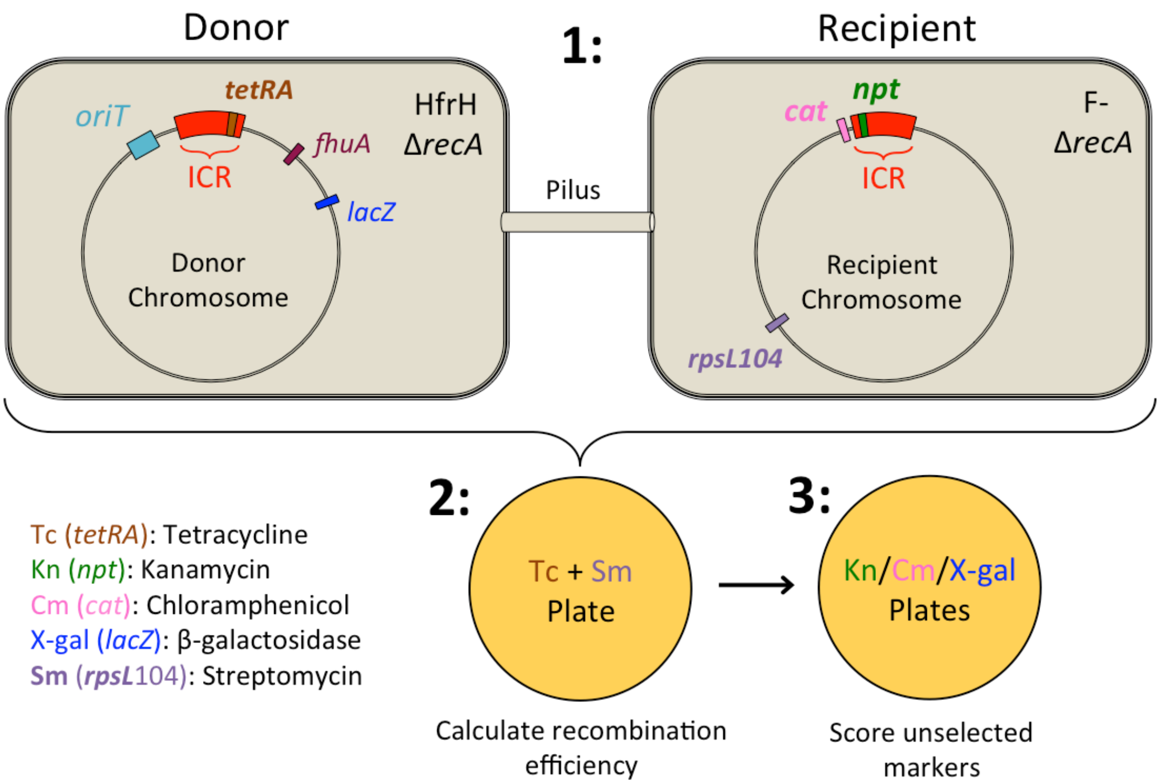

FIG S1. Model system for studying RecA-independent recombination events. 1: DNA transfer is mediated by a conjugal mating between an HfrH donor and a F^{-} recipient. Both strains are Δ*recA* to prevent homologous recombination. The donor's drug resistance (*mrr*::*tetRA*) is within the island. The recipient carries unselected drug markers in or near the ICR (*yjiT*::*npt* and *rpnD*::*cat*), *lacZ* defect ~400 Kb distal to the ICR, the selected streptomycin resistance marker (*rpsL104*) ~3 Mb distal to the ICR. During the mating, leading-strand DNA synthesis starting at *oriT* in donor displaces the complementary single strand, which is transferred to the recipient, where lagging-strand synthesis copies it. Transfer begins with *oriT* and moves clockwise through the *tetRA*-ICR construct. 2: Recombinants are selected on Tc + Sm media, with separate determinations of viable donor and recipient numbers. Recombination efficiency is calculated as the frequency at which recombinants form per recipient per mating. 3: Recombinants are scored

for the unselected markers (*npt*, *cat*, and *lacZ*) to estimate how much of the recipient genome was replaced by the incorporated donor DNA.

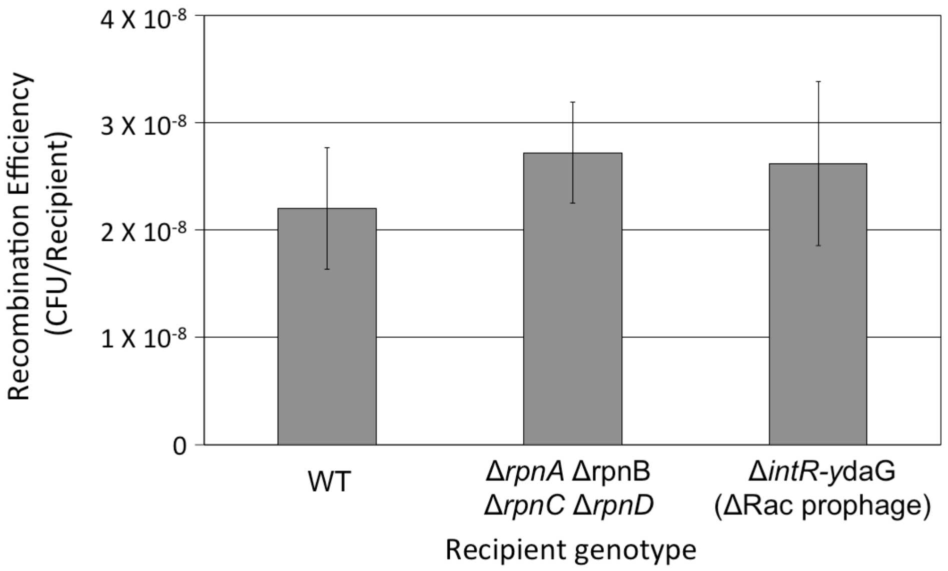

FIG S2: Basal recombination is not affected by removal of *rpn* genes or the silent recombination genes of Rac. Matings were between the Δ*recA* donor and the control recipient (WT, (ER3435 X ER3464), a derivative recipient lacking all active YhgA-like protein encoding genes (Δ*rpnA* Δ*rpnB* Δ*rpnC* Δ*rpnD;* ER3435 X ER3585) or a derivative lacking the entire Rac prophage (*ΔintR-ydaG* (ΔRac prophage), ER3613 X ER3612). Deleting the *rpn* genes or the Rac prophage had no significant effect on recombination efficiency.

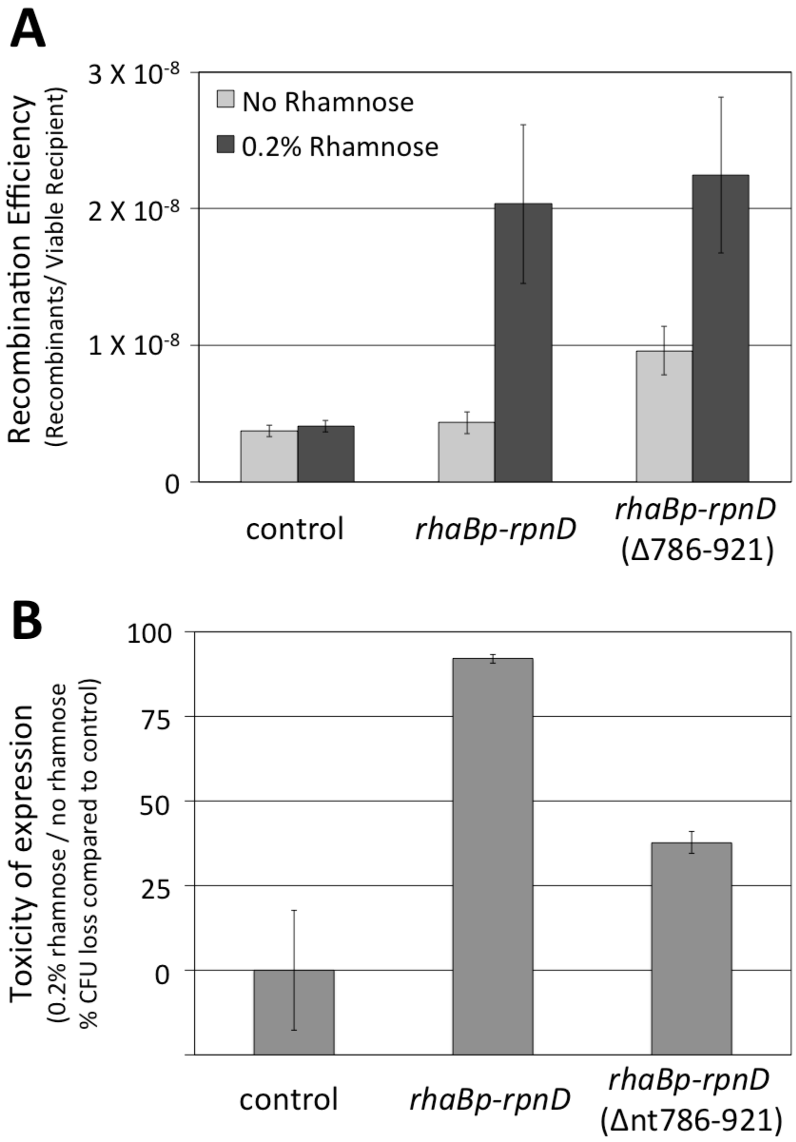

FIG S3: Removing the C-terminal tail from RpnD reduces the protein's activity. (A) The recombination efficiency of matings between the Δ*recA* donor (ER3435) and the wild type control Δ*recA* recipient (control, ER3473), or Δ*recA* recipients a with rhamnose inducible copy of either rpnD (*rhaBp-rpnD*, ER3481) or a *rpnD* variant expressing only aa 1-261 (lacking the DNA encoding its C-terminal 45 residues (*rhaBp-rpnD*(Δnt786-921), ER3559) with and without induction. Recombination efficiency is significantly higher upon induction of the shortened

RpnD variant (P-value = 0.043), but the ~2.3 fold increase is half the ~4.7 fold increase in recombination efficiency upon WT RpnD induction. (B) The effect of rhamnose on cell toxicity during these matings. The shortened RpnD variant still reduced cell viability compared to the control, but the effect was smaller than with WT RpnD.

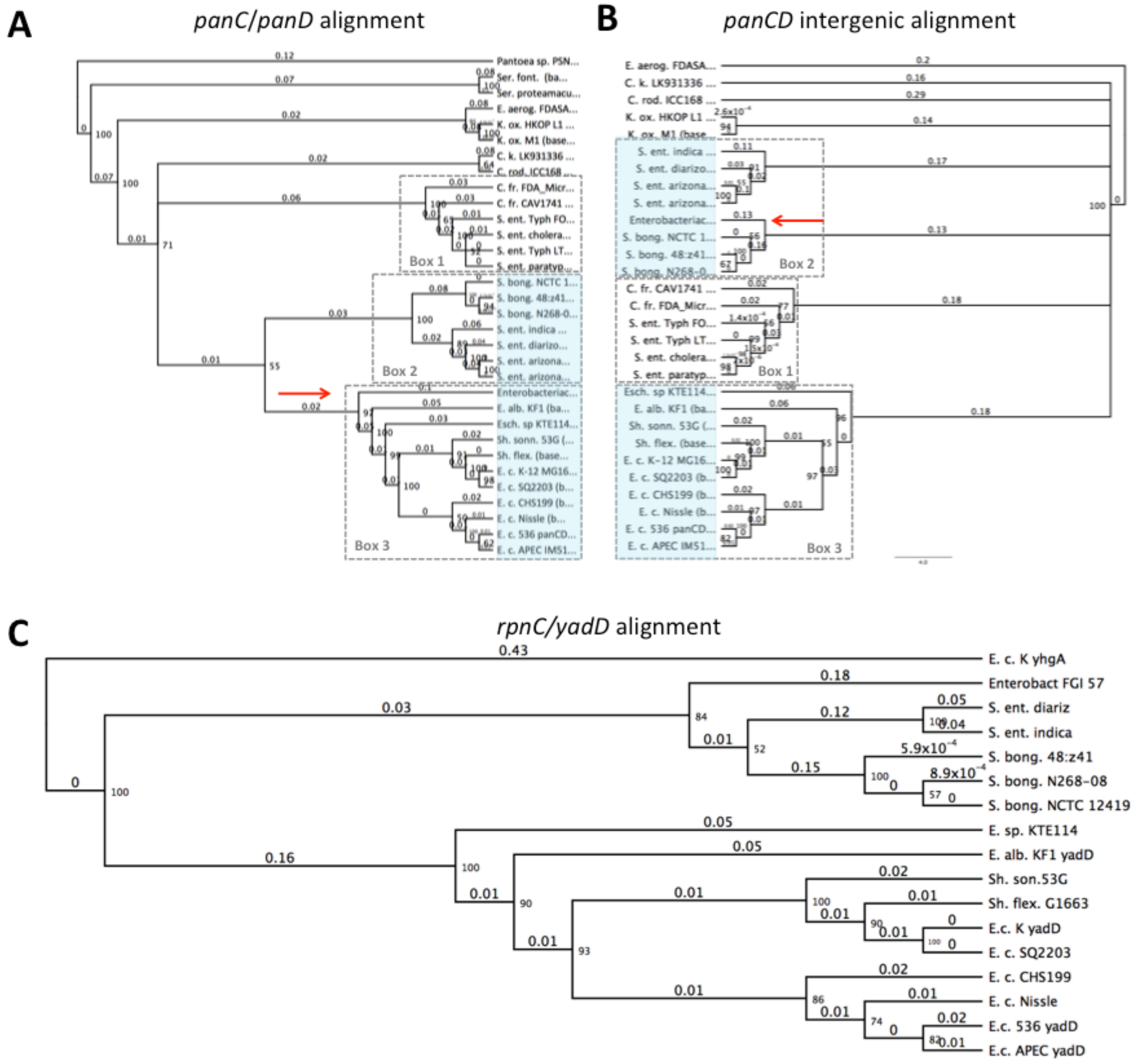

FIG S4. Phylogenetic context of the conserved *panC-panD* CDS and intergenic region. (A) *panCD* joined CDS tree. Neighbor-joining tree of a CLUSTAL alignment of catenated *panC* and *panD* homologs. Jukes-Cantor distances were used, with *Pantoea* (*Erwinia*) as an outgroup. Those strains containing *yadD* homology between *panC* and *panD* are highlighted in light blue. Nodes are labeled with the % of trees that support the node position; branches are labeled with substitutions per nucleotide along the branch. The red arrow points to the unclassified *Enterobacteriaceae* bacterium. (B) Intergenic region tree. Neighbor-

joining tree of a MAUVE alignment of the intergenic regions lying between the *panC* and *panD* genes. Three strains (*Pantoea* and *Serratia*) from the original 32-strain alignment were omitted because the short (70 or 223 nt) intergenic regions could not be aligned with the remaining sequences as part of the same Locally Colinear Block, using a 15 nt seed criterion. Labels and boxes are as in panel A. (C) *yadD* coding sequence of *Enterobacterium* FGI 57 clusters with *Salmonella* rather than *E. coli*. Annotated coding sequences with similarity to *yadD* of K-12 were extracted from *panC-panD* intergenic regions, aligned using CLUSTALW with default parameters (CLUSTALW cost matrix, a gap open cost of 15 and a gap extend cost of 6.66), and a tree built as described for panel A, using *E. coli* K-12 *rpnA* as an outgroup (top branch). Two of the 18 blue-boxed sequences in panel B were dropped because of large internal deletions in *yadD*.

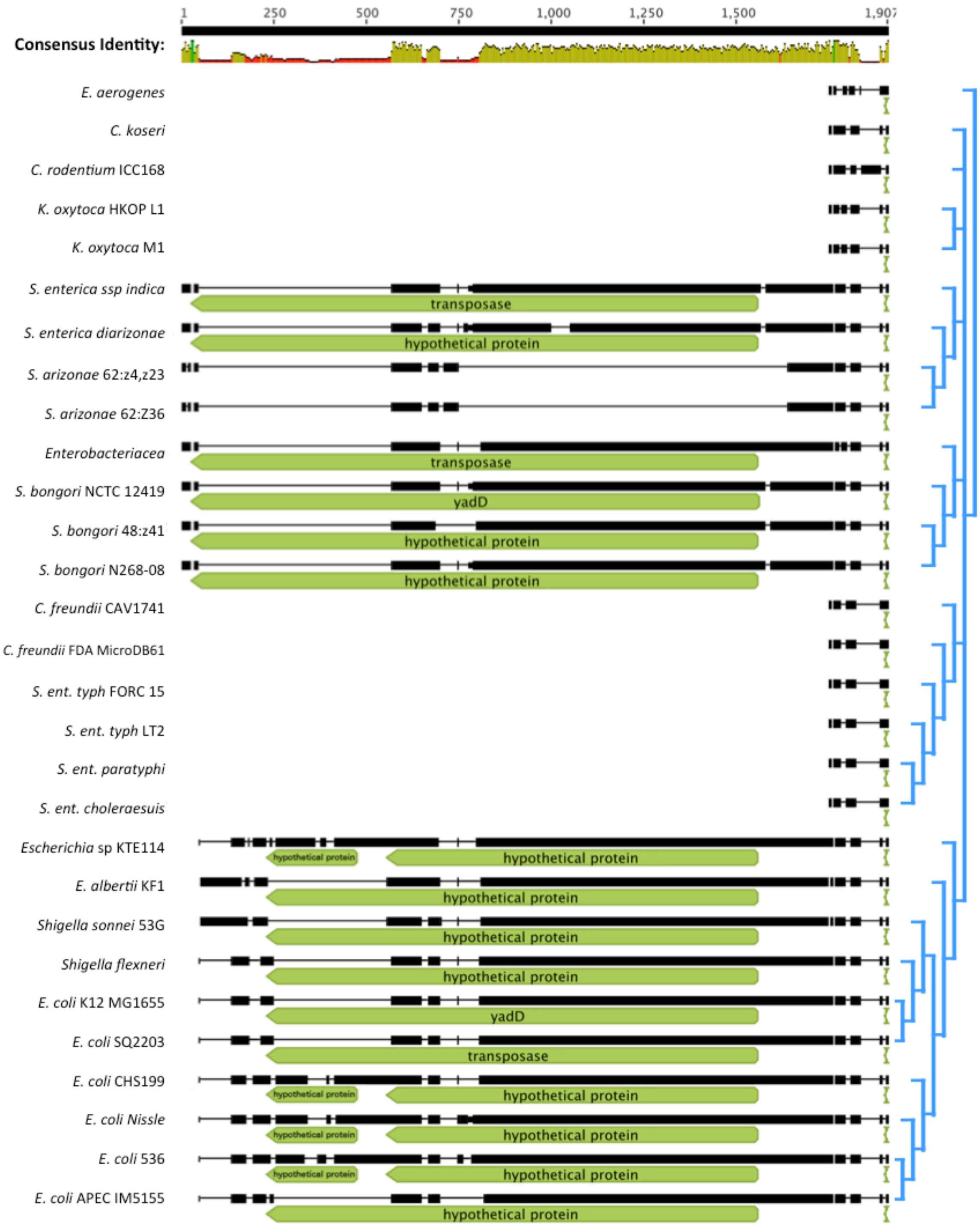

FIG S5. Mauve aligment of panCD intergenic regions among the 29 strains with alignable sequence from Table S2. The black bars denote DNA sequences aligned to the consensus with black lines signifying gaps in the sequence. Green bars represent CDS identified in the reference sequence.

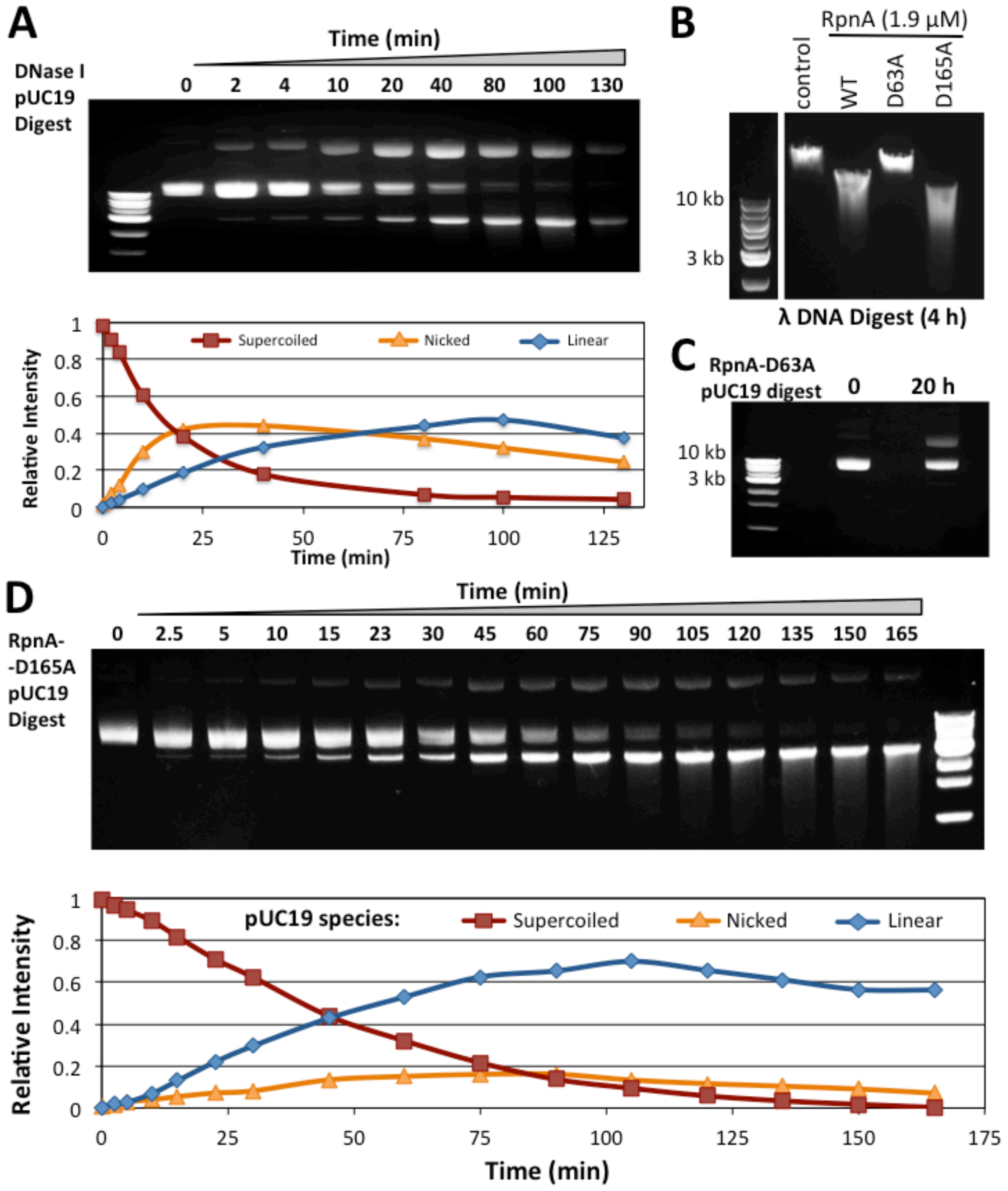

FIG S6: The kinetics of DNase I and the RpnA variants. (A) A time course assay of Dnase I activity (1.2 pM) on pUC19 (57 nM, 100 µg/mL). As expected from the known properties of DNAse I (1), the nicked species accumulates (> 40%) before substantial linearization occurs and over 90% of the supercoiled pUC19 is digested by 80 minutes by DNase I. (B) WT RpnA and the variants RpnA-D63A

and RpnA-D165A (1.9 µM, 4 h) digestion of λ DNA (3.9 nM, 125 µg/mL). RpnA degrades λ DNA without producing bands; RpnA-D63A shows no activity and RpnA-D165A exhibits more-extensive fragmentation. (C) RpnA-D63A (7.5 µM) has almost no effect on pUC19 (29 nM, 50 µg/mL) over a 20 hour reaction. (D) Time course of RpnA-D165A (7.5 µM) digestion of pUC19 (50 µg/mL) in standard RpnA buffer. Over 90% of the supercoiled pUC19 is digested within 90 minutes.

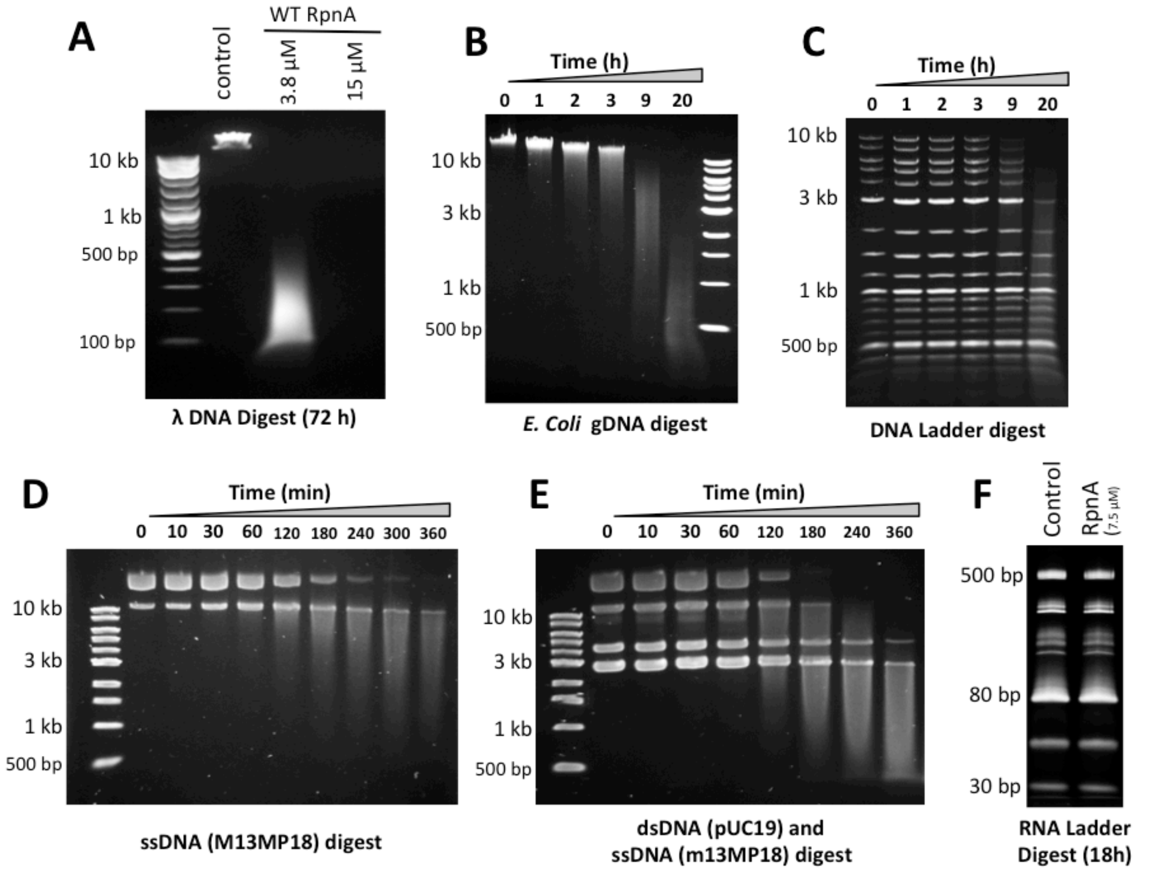

FIG S7: RpnA is a non-specific DNA endonuclease. (A) In a 72 h digest, 15 µM RpnA degrades λ DNA (7.8 nM, 250 µg/mL) to products smaller than 100 bp while 3.8 µM RpnA yields an undifferentiated smear of DNA from 100-500 bp. (B) RpnA (7.5 µM) digested *E. coli* K-12 genomic DNA (333 ug/mL) to a low MW smear. (C) When RpnA is incubated with 2-log DNA ladder (100 µg/mL), higher MW bands disappear first and no single band appears to degrade faster or slower relative to its size. (D) RpnA (3.8 µM) degrades M13MP18 ssDNA (20 nM, 50 µg/mL). (E) When RpnA (7.5 µM) added to a mixture containing M13MP18 ssDNA (20 nM, 50 µg/mL) and pUC19 dsDNA (60 nM, 100 µg/mL), both substrates are degraded at similar rates. (F) RpnA (15 µM) did not noticeably degrade double stranded RNA ladder (250 µg/mL) over an 18 hour digest.

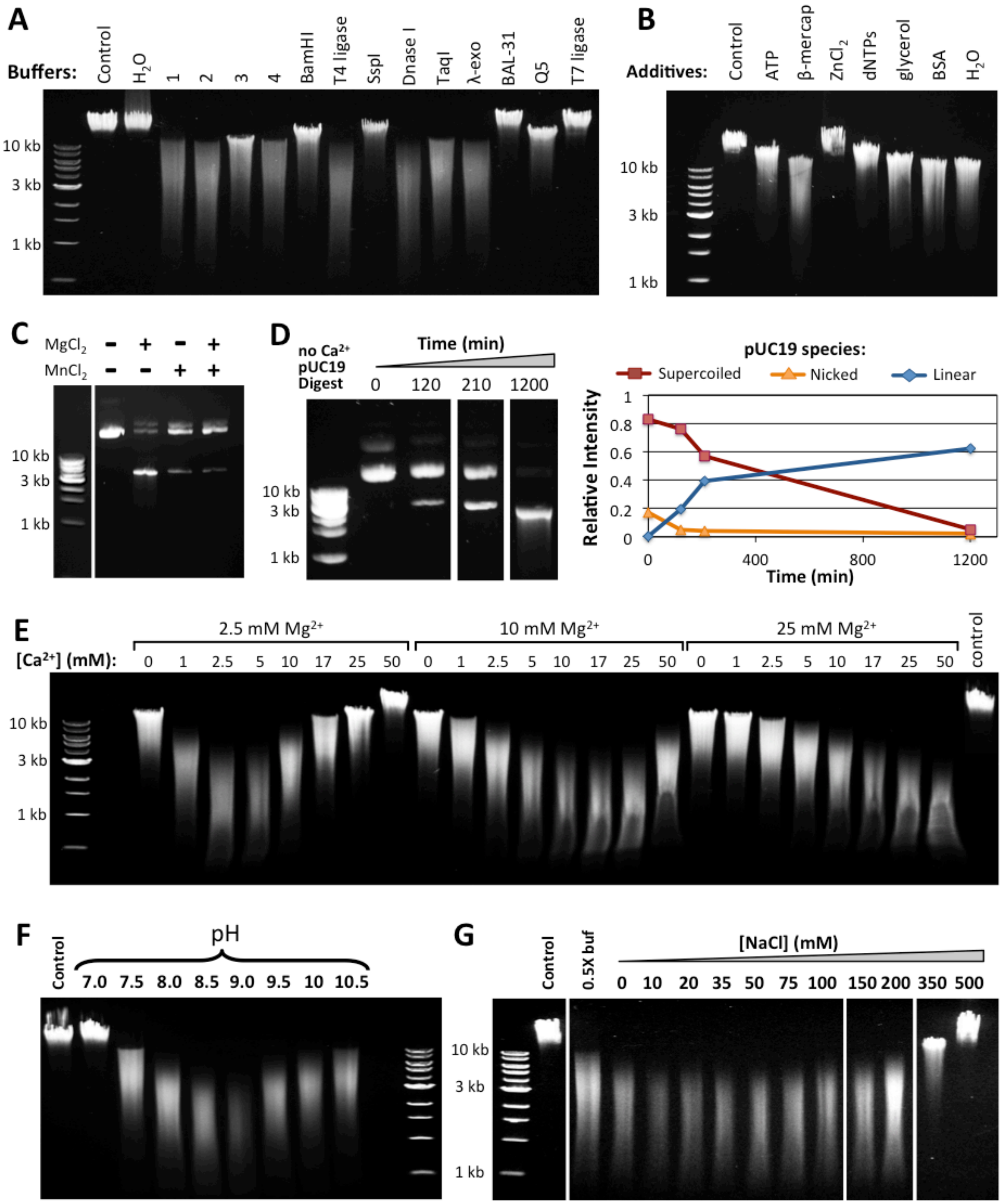

FIG S8: RpnA endonuclease buffer optimization. DNA and protein concentrations and incubation time are listed below the legend (A) Survey of commercial buffers: digestion by RpnA in various NEB buffers (compositions listed below). T4 ligase and DNaseI buffers contain supplements that affect RpnA activity (DTT, ATP, and Ca^{2+}). (B) Additive survey: RpnA in Buffer 2 with indicated additives, or

without enzyme (control) or with no additive (H_2O). (C) Divalent cations: pUC19 digested with and without 10 mM $MgCl_2$ and/or $MnCl_2$ (50 mM NaCl, 10 mM Tris pH 8.0). Mn^{2+} supports RpnA action in the absence of Mg^{2+}, but is inhibitory in its presence. (D) Kinetic analysis without Ca^{2+}: RpnA digestion of pUC19 in buffer without calcium achieves 90% linearization in 1200 min. (E) Calcium stimulation: reaction buffer with three Mg^{2+} concentrations (2.5, 10, and 25 mM) and 8 Ca^{2+} concentrations (50 mM NaCl, 10 mM Tris pH 8.0). RpnA activity was highest when [Ca^{2+}] was 1x – 2x [Mg^{2+}]. (F) pH dependence: optimum additives (50 mM NaCl, 10 mM $MgCl_2$, 10 mM $CaCl_2$, 1 mM DTT) buffered with 10 mM Tris HCl (at pH 7.0, 7.5, 8.0, 8.5, or 9.0) or sodium carbonate (at pH 9.5, 10.0, 10.5). RpnA exhibited endonuclease activity over a pH range of 7.5 -10.5 with peak activity at a pH of 9.0. (G) NaCl tolerance: optimized buffer (10 mM Tris pH 8.0, 10 mM $MgCl_2$, 10 mM $CaCl_2$, 1 mM DTT) supplemented with varying concentrations of NaCl. The “0.5X buf”: half the concentration of reaction buffer and no NaCl. RpnA was active in NaCl concentrations ranging from 0 to 200 mM with maximal activity plateauing between 10 mM and 75 mM. Based on these experiments, we defined the standard RpnA buffer to be 10 mM Tris-HCl pH 9.0, 50 mM NaCl, 10 mM $MgCl_2$, 15 mM $CaCl_2$, and 1 mM DTT to maximize the endonuclease activity of the enzyme.

Panels used the following protein concentration, incubation time, DNA substrate and DNA concentration: A, 15 μM RpnA, 24 h, λ DNA, 3.9 nM, 125 μg/mL; B, 3.8 μM RpnA, 18 h, λ DNA, 3.9 nM 125 μg/mL; C, 3.8 μM RpnA, 18 h, pUC19 ,29 nM, 50 μg/mL; D, 7.5 μM RpnA, pUC19, 29 nM, 50 μg/mL; E, 1.9 μM RpnA, 20 h,

λ DNA , 3.9 nM, 125 μg/mL; F, 1.9 μM RpnA, 20 h, λ DNA, 3.9 nM, 125 μg/mL; G, 1.9 μM RpnA, 20 h, λ DNA , 3.9 nM, 125 μg/mL.

Buffer compositions:
1: 10mM Bis-Tris-Propane-HCl, 10mM $MgCl_2$, pH 7.0@25°C
2: 50mM NaCl, 10mM Tris-HCl, 10mM $MgCl_2$, pH 7.9@25°C
3: 100mM NaCl, 50mM Tris-HCl, 10mM $MgCl_2$, 1mM DTT, pH 7.9@25°C
4: 50mM Potassium Acetate, 20mM Tris-acetate, 10mM Magnesium Acetate, 100μg/ml BSA, pH 7.9@25°C
BamHI: 150 mM NaCl, 10 mM $MgCl_2$, 10 mM Tris-HCl, 100 μg/ml BSA, 1 mM DTT, pH 7.9@25°C
T4 ligase: 50 mM Tris-HCl, 10 mM $MgCl_2$, 1 mM ATP, 10 mM DTT, pH 7.5 @ 25°C
SspI: 50 mM NaCl, 8 mM MgCl2, 8 mM 2-mercaptoethanol, 100 μg/ml BSA
DNase I: 10mM Tris-HCl, 2.5mM $MgCl_2$, 0.5mM $CaCl_2$, pH 7.6@25°C
TaqI: 100mM NaCl, 10 mM Tris-HCl, 10mM $MgCl_2$, 10 mM 2-mercaptoethanol, 100 μg/ml BSA, pH 8.4 @ 25°C
λ-exo: 67 mM Glycine-KOH, 2.5 mM $MgCl_2$, 50 μg/ml BSA, pH 9.4 @ 25°C
BAL-31 nuclease: 20 mM Tris-HCl, 600 mM NaCl, 12 mM $MgCl_2$, 12 mM $CaCl_2$, 1 mM EDTA, pH 8 @ 25°C
Q5: Data not available, Lot # 0021604.
T7 ligase: 66 mM Tris-HCl, 10 mM $MgCl_2$, 1 mM ATP, 1 mM DTT, 7.5% Polyethylene glycol (PEG 6000), pH 7.6 @ 25°C.

Text S1. Bioinformatic evidence for *rpnC*/*yadD* mobility

Identifying independent insertion events using a phylogenetic approach.

We sought to identify independent insertion events of *rpnA* or its paralogs in the genomes of strains that would help to define the mobile unit and target site characteristics. Rpn relatives are often annotated as transposases because of a suggestion made early in the genomic era. In a study of pantothenate (*pan*) biosynthesis genes, an extraneous coding sequence (initially ORF3, then *yadD*, and now *rpnC*) was found in the middle of the *pan* operon (2). Because related genes were present elsewhere in the *E. coli* K-12 MG1655 genome, the authors proposed that these represented a novel insertion sequence family. The names T_den_put_tpse (TIGRFam01784; http://www.jcvi.org/cgi-bin/tigrfams/HmmReportPage.cgi?acc=TIGR01784) and Transposase_31 (PF04754) are commonly assigned to family members. The "YhgA-like" family (PF04754; COG5464) was further defined by fold-prediction approaches (3, 4). The Interpro database (5) at present lists 5679 family members scattered among all domains including all 13 bacterial phyla, with 7 in metazoa and one in a green plant (http://www.ebi.ac.uk/interpro/entry/IPR006842/proteins-matched). Although known IS-like mobile elements are actively shaping the genomes of laboratory strains *E. coli* K-12 and B (6, 7), preliminary exploration of laboratory and wild *E. coli* isolate genomes did not yield evidence of new insertions for this family (not shown), discouraging the idea that these are IS elements.

A different model for self-mobility of *yhgA*-like genes invokes parallels with homing endonucleases: site-specific DNA recognition at a rare site in a target genome followed by repair using the invading gene as template. Target recognition function by the C-terminal protein domain would account for C-terminal variability among paralogs. Such a model predicts that genes with similar C-termini would insert at the same or similar target sites, in which case the "same" site could receive multiple independent insertions.

Thus we used a phylogenetic approach to trace the acquisition of *rpnC,* one of the paralogs studied here. Its gene location is favorable: we easily identified an unoccupied syntenic site, between *panC* and *panD,* in the other main enteric model organism, *Salmonella enterica* Typhimurium LT2. Also, because panthothenate biosynthesis is a core cellular function in enteric bacteria, conservation and synteny of the relevant genes may be anticipated across the group, facilitating sequence recovery. BLAST searches of Enterobacteriaceae represented in the NCBI Genome database (8) were used to recover exemplars of *panCD*. 32 of these were chosen to span the divergence of sequence, 18 with and 14 without an intervening segment similar to *rpnC* (Table S2). These were aligned and trees constructed as described in Methods (below).

The data are consistent with two separate introductions of *rpnC*/*yadD* into the intergenic region between *panC* and *panD*. We draw this conclusion because the flanking genes show relationships consistent with the literature for enteric bacteria, but *rpnC*/*yadD* disrupts these relationships. Figure S4A shows a tree constructed from alignments of the conserved core genes *panC* and *panD*,

excluding the intergenic region and its variable *rpnC*/*yadD* genes. *Escherichia* and *Shigella* form a coherent clade (box 3) separated from *Salmonella* and *Citrobacter* (boxes 1 and 2) with *Serratia*, *Enterobacter* and *Klebsiella* well resolved. Overall, the tree of the conserved flanking sequence resembles the result obtained using the replication origin region from a large sample of Enterobacteria (9). One uncategorized strain (*Enterobacteriaceae* bacterium strain FGI 57) was included here because of its full-length *yadD* sequence. In Figure S4B, separate alignment of the intergenic region alone disrupts the pattern. 29 strains with alignable sequence fall into similar clades (Boxes 1-3 are almost the same), but nucleotide divergence is greater, and the relative placement of the deeper nodes is lost. The unclassified bacterium *Enterobacterium* FGI 57 (red arrow) has changed position, now found in Box 2, with *S. bongori* instead of Box 3 with *Escherichia*.

Figure S4B also shows the lack of resolution of deep taxa. Disruption is due both to the introduction of *rpnC*/*yadD* and to other changes in the intergenic region (Table S2): addition of small segments on either side of the *rpnC/yadD* gene, erosion of *rpnC*/*yadD* itself, and apparent duplication of a C-terminal segment of *rpnC*/*yadD.* The *rpnC*/*yadD* coding sequence itself is responsible for much of the disruption. Excluding duplicated segments and large deletions, *rpnC*/*yadD* yields a tree with two strongly supported clusters distantly related to *rpnA* (Fig. S4C).

The ORFs downstream of *rpnC*/*yadD* in four *E. coli* appear to have arisen twice by duplication of a C-terminal portion of *rpnC*/*yadD* (Fig. S5); the

downstream ORFs were not included in the analysis shown in Fig. S4C. Inclusion of those segments did not change the overall result; similar clusters were obtained (not shown), particularly the separation of the *Salmonella* and *Escherichia* groups and the position of *Enterobacterium* FGI 57.

An alternative model would require a single insertion event followed by multiple loss events together with accelerated *rpnC*/*yadD*-specific divergence tending to drive one *rpnC*/*yadD* allele to become more similar to that of a strain belonging to a cluster to which its own *panC* and *panD* genes do not belong. A hotspot for homologous recombination or localized mutagenesis could account for this pattern. A third possibility is episodic diversifying selection acting on nearby genes, such that the allele configuration containing *panC*-*yadD*-*panD* would often be imported from distant relatives. Nearby genome islands, which carry annotations relating to fimbriae, are candidates for such selection. However, it is hard to account for conservation of *panC*-*panD* distinct from *yadD* in such an eventuality.

Methods: Selection of representatives. We identified *Enterobacteriacea panCD* segments in the NCBI Genomes section with BLAST similarity to K-12 at the 5’ end of *panC* and the 3’ end of *panD*. We chose 32 of these (see Table S2) at various levels of identity with preference to representatives carrying *rpnC*/*yadD*. Preference was given to segments from complete genomes. The thousands of *E. coli/Shigella* sequences were narrowed to selected variable examples by eye.

Alignment. Continuous DNA sequence segments lying between the 5' end of the annotated CDS homologous to *panC* and the 3' end of the annotated CDS

homologous to *panD* of MG1655 were extracted to a file of files. These files were aligned using the Geneious implementation of ProgressiveMauve (10, 11) with default options and the Gapped Aligner MUSCLE 3.6 (12). This approach conserves alignment gaps at the position of the *rpnC*/*yadD* CDS (Figure S5). The *panC* and *panD* genes were extracted separately, from this alignment, selecting the segments aligning with *E. coli* MG1655 annotated CDS, then each *panC* catenated to the respective *panD*. A CLUSTALW alignment with CLUSTAL Distance was generated for the CDS alignments. Trees were made using the Geneious Tree Builder with a Juke-Cantor Genetic distance model, and a Neighbor-Joining tree build method. *Pantoea* sp was the outgroup; 1000 bootstrap resamplings with 50% support threshhold were used to create a consensus tree. Pairwise alignment similarity ranged from 30-100% identical over the complete sequence set, with *panC* and *panD* homology higher than the intergenic region.

Table S1. Strains, plasmids, and oligonucleotides used in this study.

Strain	F status	genotype[a]	plasmid	Source, reference, construction
ER1370	-	*fhuA2*::IS2 Δ(*lacZ*)*4826 glnX44 e14- trpE31* Δ(*hisG*)*1 argG6*(*FS*) *rpsL104*(*StrR*) *xyl-7 mtlA2*(*Fs*) *metB1*(*FS*) *serB28*		This lab (13)
ER1636	-	*fhuA2*::IS2 Δ(*lacZ*)*4826 glnX44 trpE31* Δ(*hisG*)*1 recD1014 rpsL104*(*StrR*) *xyl-7 mtlA2*(*Fs*) *metB1*(*FS*) *serB28*		This lab (14)
ER2170	-	*Δ(argF-lac)U169 glnX44 mcr-67 rfbD1? relA1? endA1 spoT1? dinD2::MudI1734 (KanR, lacZ(ts)) thi-1 Δ([fimB or yjiT]-opgB)114::IS10*		New England Biolabs (15)
ER2566	-	*fhuA2::IS2 lacZ::T7 gene1 [lon] ompT gal sulA11 Δ(yjiT-opgB)114::IS10 R(mcr-73::miniTn10--TetS)2 R(zgb-210::Tn10 --TetS) endA1 [dcm]*		New England Biolabs (16)
ER3252	-	*Δ*(*araBAD*)*567 ΔlacZ4787*(::*rrnB3*) *ΔrecA*::FRT::*npt*(*KnR*) *rph-1 Δ*(*rhaBAD*)*568 hsdR514*		Susan Lovett (17)
ER3340	-	*fhuA2*::IS2 Δ(*lacZ*)*4826 glnX44 trpE31* Δ(*hisG*)*1 gyrA96 recA13 recD1014 rpsL104*(*StrR*) *xyl-7 mtlA2*(*Fs*) *mTn7*(Φ (*rhaBp-lacZ*)) *metB1*(*FS*) Δ(*yjiT-mrr*)::FRT::*npt*(*KnR*) *serB28*		This lab (14)
ER3435	Hfr(PO1)[b]	*supQ80 thiE1 mrr*::*tetAR e14-* (*McrA-*) *ΔrecA*::FRT *relA1 spoT1*		This lab (14)
ER3467	-	*fhuA2*::IS2 Δ(*lacZ*)*4826 glnX44 trpE31* Δ(*hisG*)*1 recA*::FRT *recD1014 rpsL104*(*StrR*) *xyl-7 mtlA2*(*Fs*) *metB1*(*FS*) *serB28*	pKD46	This lab (14)
ER3473	-	*fhuA2*::IS2 Δ(*lacZ*)*4826 glnX44 trpE31* Δ(*hisG*)*1 recA*::FRT *recD1014 rpsL104*(*StrR*) *xyl-7 mtlA2*(*Fs*) *metB1*(*FS*) *rpnD*::*cat*(FRT) *yjiT*::*npt*(FRT) *serB28*		This lab (14)
ER3481	-	*fhuA2*::IS2 Δ(*lacZ*)*4826 glnX44 trpE31* Δ(*hisG*)*1 recD1014 rpsL104*(*StrR*) *xyl-7 mtlA2*(*Fs*) *mTn7*(Φ(*rhaBp-rpnDc*)[c] *metB1*(*FS*) *serB28 recA*::FRT *yjiT*::*npt*(FRT) *rpnD*::*cat*(FRT)		This lab (14)
ER3511	-	*fhuA2*::IS2 Δ(*lacZ*)*4826 glnX44 trpE31* Δ(*hisG*)*1 recD1014 rpsL104*(*StrR*) *xyl-7 mtlA2*(*Fs*) *mTn7*(Φ(*rhaBp-rpnB*) *metB1*(*FS*) *serB28 recA*::FRT *yjiT*::*npt*(FRT) *rpnD*::*cat*(FRT)		ER3473 X pTK014 → ApR @ 37 ^{o}C → SmR @ 42 ^{o}C x2 purifications → screened for insertion at *attTn7*
ER3512	-	*fhuA2*::IS2 Δ(*lacZ*)*4826 glnX44 trpE31* Δ(*hisG*)*1 recD1014 rpsL104*(*StrR*) *xyl-7 mtlA2*(*Fs*) *mTn7*(Φ(*rhaBp-rpnC*) *metB1*(*FS*) *serB28 recA*::FRT *yjiT*::*npt*(FRT) *rpnD*::*cat*(FRT)		ER3473 X pTK015 → ApR @ 37 ^{o}C → SmR @ 42 ^{o}C x2 purifications → screened for insertion at *attTn7*
ER3513	-	*fhuA2*::IS2 Δ(*lacZ*)*4826 glnX44 trpE31* Δ(*hisG*)*1 recD1014 rpsL104*(*StrR*) *xyl-7 mtlA2*(*Fs*) *mTn7*(Φ(*rhaBp-rpnE*) *metB1*(*FS*) *serB28 recA*::FRT *yjiT*::*npt*(FRT) *rpnD*::*cat*(FRT)		ER3473 X pTK016 → ApR @ 37 ^{o}C → SmR @ 42 ^{o}C x2 purifications → screened for insertion at *attTn7*
ER3514	-	*fhuA2*::IS2 Δ(*lacZ*)*4826 glnX44 trpE31* Δ(*hisG*)*1 recD1014 rpsL104*(*StrR*) *xyl-7 mtlA2*(*Fs*) *mTn7*(Φ(*rhaBp-rpnA*) *metB1*(*FS*) *serB28 recA*::FRT *yjiT*::*npt*(FRT) *rpnD*::*cat*(FRT)		ER3473 X pTK017 → ApR @ 37 ^{o}C → SmR @ 42 ^{o}C x2 purifications → screened for insertion at *attTn7*
ER3526	-	*fhuA2*::IS2 Δ(*lacZ*)*4826 glnX44 trpE31* Δ(*hisG*)*1 recD1014 rpsL104*(*StrR*) *xyl-7 mtlA2*(*Fs*) *mTn7*(Φ(*rhaBp-rpnB*) *metB1*(*FS*) *serB28 recA*::FRT		ER3464 X pTK014 → ApR @ 37 ^{o}C → SmR @ 42 ^{o}C x2 purifications → screened for insertion at *attTn7*
ER3527	-	*fhuA2*::IS2 Δ(*lacZ*)*4826 glnX44 trpE31* Δ(*hisG*)*1 recD1014 rpsL104*(*StrR*) *xyl-7 mtlA2*(*Fs*) *mTn7*(Φ(*rhaBp-rpnDc*)[c] *metB1*(*FS*) *serB28 recA*::FRT		R3464 X pER452 → ApR @ 37 ^{o}C → SmR @ 42 ^{o}C x2 purifications → screened for insertion at *attTn7*
ER3533	-	*fhuA2*::IS2 Δ(*lacZ*)*4826 glnX44 trpE31* Δ(*hisG*)*1 recD1014 rpsL104*(*StrR*) *xyl-7 mtlA2*(*Fs*) *mTn7*(Φ(*rhaBp-mcrA*) *metB1*(*FS*) *serB28 recA*::FRT *yjiT*::*npt*(FRT) *rpnD*::*cat*(FRT)		ER3473 X pTK022 → ApR @ 37 ^{o}C → SmR @ 42 ^{o}C x2 purifications → screened for insertion at *attTn7*
ER3535	-	*fhuA2*::IS2 Δ(*lacZ*)*4826 glnX44 trpE31* Δ(*hisG*)*1 recD1014 rpsL104*(*StrR*) *xyl-7 mtlA2*(*Fs*) *mTn7*(Φ(*rhaBp-asiSI*) *metB1*(*FS*) *serB28 recA*::FRT *yjiT*::*npt*(FRT) *rpnD*::*cat*(FRT)		ER3473 X pTK023 → ApR @ 37 ^{o}C → SmR @ 42 ^{o}C x2 purifications → screened for insertion at *attTn7*
ER3541	-	*fhuA2*::IS2 Δ(*lacZ*)*4826 glnX44 trpE31* Δ(*hisG*)*1 recD1014 rpsL104*(*StrR*) *xyl-7 mtlA2*(*Fs*) *mTn7*(Φ(*rhaBp-nt.bsrDIB*) *metB1*(*FS*) *serB28 recA*::FRT *yjiT*::*npt*(FRT) *rpnD*::*cat*(FRT)		ER3473 X pTK025 → ApR @ 37 ^{o}C → SmR @ 42 ^{o}C x2 purifications → screened for insertion at *attTn7*
ER3552	-	*fhuA2*::IS2 Δ(*lacZ*)*4826 glnX44 trpE31* Δ(*hisG*)*1 recD1014 rpsL104*(*StrR*) *xyl-7 mtlA2*(*Fs*) *mTn7*(Φ(*rhaBp-rpnA(D11A)*) *metB1*(*FS*) *serB28 recA*::FRT *yjiT*::*npt*(FRT) *rpnD*::*cat*(FRT)		ER3473 X pTK026 → ApR @ 37 ^{o}C → SmR @ 42 ^{o}C x2 purifications → screened for insertion at *attTn7*
ER3553	-	*fhuA2*::IS2 Δ(*lacZ*)*4826 glnX44 trpE31* Δ(*hisG*)*1 recD1014*		ER3473 X pTK027 → ApR @ 37 ^{o}C

		rpsL104(*StrR*) *xyl-7 mtlA2*(*Fs*) *mTn7*(Φ(*rhaBp-rpnA(D63A)*) *metB1*(*FS*) *serB28 recA*::FRT *yjiT*::*npt*(FRT) *rpnD*::*cat*(FRT)		→ SmR @ 42 °C x2 purifications → screened for insertion at *attTn7*
ER3554	-	*fhuA2*::IS2 Δ(*lacZ*)*4826 glnX44 trpE31* Δ(*hisG*)*1 recD1014 rpsL104*(*StrR*) *xyl-7 mtlA2*(*Fs*) *mTn7*(Φ(*rhaBp-rpnA(E82A)*) *metB1*(*FS*) *serB28 recA*::FRT *yjiT*::*npt*(FRT) *rpnD*::*cat*(FRT)		ER3473 X pTK028 → ApR @ 37 °C → SmR @ 42 °C x2 purifications → screened for insertion at *attTn7*
ER3555	-	*fhuA2*::IS2 Δ(*lacZ*)*4826 glnX44 trpE31* Δ(*hisG*)*1 recD1014 rpsL104*(*StrR*) *xyl-7 mtlA2*(*Fs*) *mTn7*(Φ(*rhaBp-rpnA(Q84K)*) *metB1*(*FS*) *serB28 recA*::FRT *yjiT*::*npt*(FRT) *rpnD*::*cat*(FRT)		ER3473 X pTK029 → ApR @ 37 °C → SmR @ 42 °C x2 purifications → screened for insertion at *attTn7*
ER3556	-	*fhuA2*::IS2 Δ(*lacZ*)*4826 glnX44 trpE31* Δ(*hisG*)*1 recD1014 rpsL104*(*StrR*) *xyl-7 mtlA2*(*Fs*) *mTn7*(Φ(*rhaBp-rpnA(Q84A)*) *metB1*(*FS*) *serB28 recA*::FRT *yjiT*::*npt*(FRT) *rpnD*::*cat*(FRT)		ER3473 X pTK030 → ApR @ 37 °C → SmR @ 42 °C x2 purifications → screened for insertion at *attTn7*
ER3557	-	*fhuA2*::IS2 Δ(*lacZ*)*4826 glnX44 trpE31* Δ(*hisG*)*1 recD1014 rpsL104*(*StrR*) *xyl-7 mtlA2*(*Fs*) *mTn7*(Φ(*rhaBp-rpnA(R94A)*) *metB1*(*FS*) *serB28 recA*::FRT *yjiT*::*npt*(FRT) *rpnD*::*cat*(FRT)		ER3473 X pTK031 → ApR @ 37 °C → SmR @ 42 °C x2 purifications → screened for insertion at *attTn7*
ER3558	-	*fhuA2*::IS2 Δ(*lacZ*)*4826 glnX44 trpE31* Δ(*hisG*)*1 recD1014 rpsL104*(*StrR*) *xyl-7 mtlA2*(*Fs*) *mTn7*(Φ(*rhaBp-rpnA(D165A)*) *metB1*(*FS*) *serB28 recA*::FRT *yjiT*::*npt*(FRT) *rpnD*::*cat*(FRT)		ER3473 X pTK032 → ApR @ 37 °C → SmR @ 42 °C x2 purifications → screened for insertion at *attTn7*
ER3559	-	*fhuA2*::IS2 Δ(*lacZ*)*4826 glnX44 trpE31* Δ(*hisG*)*1 recD1014 rpsL104*(*StrR*) *xyl-7 mtlA2*(*Fs*) *mTn7*(Φ(*rhaBp-rpnD*(Δ786-921)) *metB1*(*FS*) *serB28 recA*::FRT *yjiT*::*npt*(FRT) *rpnD*::*cat*(FRT)		ER3473 X pTK033 → ApR @ 37 °C → SmR @ 42 °C x2 purifications → screened for insertion at *attTn7*
ER3560	-	*Δ(argF-lac)U169 glnX44 mcr-67 rfbD1? relA1? endA1 spoT1? dinD2::MudI1734 (KanR, lacZ(ts)) thi-1 Δ([fimB or yjiT]-opgB)114::IS10*	pER452	ER2170 X pER452 → ApR @ 30 °C
ER3561	-	*Δ(argF-lac)U169 glnX44 mcr-67 rfbD1? relA1? endA1 spoT1? dinD2::MudI1734 (KanR, lacZ(ts)) thi-1 Δ([fimB or yjiT]-opgB)114::IS10*	pTK014	ER2170 X pTK014 → ApR @ 30 °C
ER3562	-	*Δ(argF-lac)U169 glnX44 mcr-67 rfbD1? relA1? endA1 spoT1? dinD2::MudI1734 (KanR, lacZ(ts)) thi-1 Δ([fimB or yjiT]-opgB)114::IS10*	pTK015	ER2170 X pTK015 → ApR @ 30 °C
ER3563	-	*Δ(argF-lac)U169 glnX44 mcr-67 rfbD1? relA1? endA1 spoT1? dinD2::MudI1734 (KanR, lacZ(ts)) thi-1 Δ([fimB or yjiT]-opgB)114::IS10*	pTK016	ER2170 X pTK016 → ApR @ 30 °C
ER3564	-	*Δ(argF-lac)U169 glnX44 mcr-67 rfbD1? relA1? endA1 spoT1? dinD2::MudI1734 (KanR, lacZ(ts)) thi-1 Δ([fimB or yjiT]-opgB)114::IS10*	pTK017	ER2170 X pTK017 → ApR @ 30 °C
ER3569	-	*fhuA2*::IS2 Δ(*lacZ*)*4826 glnX44 trpE31* Δ(*hisG*)*1 recD1014 rpsL104*(*StrR*) *xyl-7* Δ*rpnA mtlA2*(*Fs*) *metB1*(*FS*) *serB28*		ER1636 X pTK034 → KnR @ 37 °C → SIR media → Screened for *rpnA* deletion
ER3570	-	*fhuA2*::IS2 Δ(*lacZ*)*4826 glnX44 trpE31* Δ(*hisG*)*1* Δ*rpnB recD1014 rpsL104*(*StrR*) *xyl-7* Δ*rpnA mtlA2*(*Fs*) *metB1*(*FS*) *serB28*		ER1636 X pTK035 → KnR @ 37 °C → SIR media → Screened for *rpnB* deletion
ER3571	-	*fhuA2*::IS2 Δ*rpnC* Δ(*lacZ*)*4826 glnX44 trpE31* Δ(*hisG*)*1* Δ*rpnB recD1014 rpsL104*(*StrR*) *xyl-7* Δ*rpnA mtlA2*(*Fs*) *metB1*(*FS*) *serB28*		ER3570 X pTK036 → KnR @ 37 °C → SIR media → Screened for *rpnC* deletion
ER3572	-	*fhuA2*::IS2 Δ*rpnD* Δ*rpnC* Δ(*lacZ*)*4826 glnX44 trpE31* Δ(*hisG*)*1* Δ*rpnB recD1014 rpsL104*(*StrR*) *xyl-7* Δ*rpnA mtlA2*(*Fs*) *metB1*(*FS*) *serB28*		ER3571 X pTK037 → KnR @ 37 °C → SIR media → Screened for *rpnD* deletion
ER3573	-	*fhuA2::IS2 lacZ::T7 gene1 [lon] ompT gal sulA11 Δ(yjiT-opgB)114::IS10 R(mcr-73::miniTn10--TetS)2 R(zgb-210::Tn10 --TetS) endA1 [dcm]*	pTK038	ER2566 X pTK038 → ApR @ 37 °C
ER3577	-	*Δ(argF-lac)U169 glnX44 mcr-67 rfbD1? relA1? endA1 spoT1? dinD2::MudI1734 (KanR, lacZ(ts)) thi-1 Δ([fimB or yjiT]-opgB)114::IS10*	pTK026	ER2170 X pTK026 → ApR @ 30 °C
ER3578	-	*Δ(argF-lac)U169 glnX44 mcr-67 rfbD1? relA1? endA1 spoT1? dinD2::MudI1734 (KanR, lacZ(ts)) thi-1 Δ([fimB or yjiT]-opgB)114::IS10*	pTK027	ER2170 X pTK027 → ApR @ 30 °C
ER3579	-	*Δ(argF-lac)U169 glnX44 mcr-67 rfbD1? relA1? endA1 spoT1? dinD2::MudI1734 (KanR, lacZ(ts)) thi-1 Δ([fimB or yjiT]-*	pTK028	ER2170 X pTK028 → ApR @ 30 °C

		opgB)114::IS10		
ER3580	-	*Δ(argF-lac)U169 glnX44 mcr-67 rfbD1? relA1? endA1 spoT1? dinD2::MudI1734 (KanR, lacZ(ts)) thi-1 Δ([fimB or yjiT]-opgB)114::IS10*	pTK029	ER2170 X pTK029 → ApR @ 30 °C
ER3581	-	*Δ(argF-lac)U169 glnX44 mcr-67 rfbD1? relA1? endA1 spoT1? dinD2::MudI1734 (KanR, lacZ(ts)) thi-1 Δ([fimB or yjiT]-opgB)114::IS10*	pTK030	ER2170 X pTK030 → ApR @ 30 °C
ER3582	-	*Δ(argF-lac)U169 glnX44 mcr-67 rfbD1? relA1? endA1 spoT1? dinD2::MudI1734 (KanR, lacZ(ts)) thi-1 Δ([fimB or yjiT]-opgB)114::IS10*	pTK031	ER2170 X pTK031 → ApR @ 30 °C
ER3583	-	*Δ(argF-lac)U169 glnX44 mcr-67 rfbD1? relA1? endA1 spoT1? dinD2::MudI1734 (KanR, lacZ(ts)) thi-1 Δ([fimB or yjiT]-opgB)114::IS10*	pTK032	ER2170 X pTK032 → ApR @ 30 °C
ER3585	-	*fhuA2*::IS2 *ΔrpnD ΔrpnC* Δ(*lacZ*)*4826 glnX44 trpE31* Δ(*hisG*)*1 recA*::*kan ΔrpnB recD1014 rpsL104*(*StrR*) *xyl-7 ΔrpnA mtlA2*(*Fs*) *metB1*(*FS*) *serB28*		ER3572 X P1*vir*(ER3252) → KnR
ER3609	-	*fhuA2::IS2 lacZ::T7 gene1 [lon] ompT gal sulA11 Δ(yjiT-opgB)114::IS10 R(mcr-73::miniTn10--TetS)2 R(zgb-210::Tn10 --TetS) endA1 [dcm]*	pTK043	ER2566 X pTK043 → ApR @ 37 °C
ER3610	-	*fhuA2::IS2 lacZ::T7 gene1 [lon] ompT gal sulA11 Δ(yjiT-opgB)114::IS10 R(mcr-73::miniTn10--TetS)2 R(zgb-210::Tn10 --TetS) endA1 [dcm]*	pTK044	ER2566 X pTK044 → ApR @ 37 °C
ER3611	Hfr(PO1)[b]	*supQ80 thiE1 e14-* (*McrA-*) *ΔrecA*::FRT *relA1 spoT1 mrr*::*tetAR*	pKD46	ER3435 X pKD46 → ApR TcR @ 30 °C
ER3612	-	*fhuA2*::IS2 Δ(*lacZ*)*4826 glnX44 trpE31 ttcA-C::cat*(ΔRac) Δ(*hisG*)*1 recA*::FRT *recD1014 rpsL104*(*StrR*) *xyl-7 mtlA2*(*Fs*) *metB1*(*FS*) *serB28*		ER3467 X *ttcA*-P1-*cat*-P2-*ttcC* PCR product → CmR @ 37 °C → CmR + SmR @ 42 °C x2 purifications → Screened for loss of Rac prophage
ER3613	Hfr(PO1)[b]	*supQ80 thiE1 mrr*::*tetAR e14-* (*McrA-*) *ttcA-C::cat*(ΔRac) *ΔrecA*::FRT *relA1 spoT1*		ER3611 X *ttcA*-P1-*cat*-P2-*ttcC* PCR product → CmR @ 37 °C → CmR + Tc @ 42 °C x2 purifications → Screened for lRac prophage

[a]All strains in this list are derived from *E. coli* K1-2
[b]The full genotype of the Hfr(PO1) element is Hfr(PO1) (*Δ(pifA-yddA)* Δ(*ybiB-yfgA*)::FRT)
[C]*rpnD* in *E. coli* K-12 has a premature stop codon. All *rpnD* overexpression experiments were performed with a mutated K-12 *rpnD* lacking the premature stop codon (*rpnDc*, for *rpnDcomplete*)

Vector	Genotype	Source
pMS34	*oripSC101ts ApR araBp*::*TnsABCD araCp-araC mTn7*(Φ(*rhaBp-lacZ*)	(18)
pCP20	*ts-rep* [cI857](λ)(ts) *bla*(ApR) *cat* FLP([])	(19)
pKD46	*repA101*(*ts*) *araBp-gam-bet-exo oriR101 bla*(ApR) *araC bla* [tL3]	(19)
pKD4	*oriR*(R6Kγ) *bla*(AmpR) *rgnB*(Ter) *npt*(KnR)	(19)
pKD32	*oriR*(R6Kγ) *tL3*λ(*ter*) *bla*(AmpR) *rgnB*(Ter) *cat*(CamR)	(19)
pER452	PMS34 *mTn7*(Φ(*rhaBp-rpnD*)	(14)
pCD2	*lac*(α)::*mrr*::(*tetAtetR*) *cat* (CmR) *rep*(*pSC101ts*)	(14)
pTXB1	*bla lacI* pTN7	NEB[a]
pDEL-R6K	ori(γ-R6K) (rhaBp-I-SceI) Kan (Ptac-sacB)	(20)
pTK014	PMS34 *mTn7*(Φ(*rhaBp-rpnB*)	This work
pTK015	PMS34 *mTn7*(Φ(*rhaBp-rpnC*)	This work
pTK016	PMS34 *mTn7*(Φ(*rhaBp-rpnE*)	This work
pTK017	PMS34 *mTn7*(Φ(*rhaBp-rpnA*)	This work

pTK022	PMS34 *mTn7*(Φ(*rhaBp-mcrA*)	This work
pTK023	PMS34 *mTn7*(Φ(*rhaBp-asiSI*)	This work
pTK025	PMS34 *mTn7*(Φ(*rhaBp-Nt.BsrDIB*)	This work
pTK026	PMS34 *mTn7*(Φ(*rhaBp-rpnA(D11A)*)	This work
pTK027	PMS34 *mTn7*(Φ(*rhaBp-rpnA(D63A)*)	This work
pTK028	PMS34 *mTn7*(Φ(*rhaBp-rpnA(E82A)*)	This work
pTK029	PMS34 *mTn7*(Φ(*rhaBp-rpnA(Q84K)*)	This work
pTK030	PMS34 *mTn7*(Φ(*rhaBp-rpnA(Q84A)*)	This work
pTK031	PMS34 *mTn7*(Φ(*rhaBp-rpnA(R94A)*)	This work
pTK032	PMS34 *mTn7*(Φ(*rhaBp-rpnA(D165A)*)	This work
pTK032	PMS34 *mTn7*(Φ(*rhaBp-rpnD*(Δ786-921))	This work
pTK034	pDEL-R6K Δ*rpnA*	This work
pTK035	pDEL-R6K Δ*rpnB*	This work
pTK036	pDEL-R6K Δ*rpnC*	This work
pTK037	pDEL-R6K Δ*rpnD*	This work
pTK038	*bla lacI* pTN7-*rpnA*	This work
pTK043	*bla lacI* pTN7-*rpnA*(D63A)	This work
pTK044	*bla lacI* pTN7-*rpnA*(D165A)	This work

[a] See The 2015-16 NEB Catalog & Technical Reference or visit the NEB website (www.neb.com) for a full description of NEB plasmids.

Oligo #	Name	Sequence
pER91	pMAKmrrright	GCTCGGTACCCGGGGATCCAGGCCGTGGGGCGATGAAA
pER92	pMAKmrrleft	TACGCCAAGCTTGCATGCCATTGCTGTGCGGGCCTGTCC
oTK057	pMS34-rpnC up	ACACAGGAGGGACGTATGGATGCACCGAGTACC
oTK058	rpnC do-pMS34	CCAGCGGCCGCGTTTTACCCCACGTCATCTGTC
oTK059	pMS34-rpnB up	ACACAGGAGGGACGTATGACAATATCGACAACTTCC
oTK060	rpnB do-pMS34	CCAGCGGCCGCGTTTTAACTCCCGGTGTTACAG
oTK061	pMS34-rpnE up	ACACAGGAGGGACGTATGACCGAATCAACAACC
oTK062	rpnE do-pMS34	CCAGCGGCCGCGTTTTCCTTTTAATGACAGTTCGG
oTK063	pMS34-yghA up	ACACAGGAGGGACGTATGAGCAAAAAGCAGAGTTCC
oTK064	rpnA do-pMS34	CCAGCGGCCGCGTTTTTACTGACTCGCCGCAGC
oTK078	pMS34-mcrA up	ACACAGGAGGGACGTATGCATGTTTTTGATAATAATGGA
oTK079	pMS34-mcrA do	CCAGCGGCCGCGTTTTTACCCTGTAGGGTATTACGTCTT
oTK080	pMS34-asiSI up	ACACAGGAGGGACGTATGGGCGAGTCTATTGATCAA
oTK081	pMS34-asiSI do	CCAGCGGCCGCGTTTTCCAACAGTCCGGTCGATA
oTK086	pDEL-R6K_rev	CGTCGTGAATTACCATGTCAGCCG
oTK087	pDEL-R6K_fwd	GCCACCTCATGTTAGTCATGCGC
oTK088	rpnC_UP_fwd	CATGGTAATTCACGACGAACATTGTCGCGGCGCAG
oTK089	rpnC_UP_rev	AGCCATGAGAAAAAGATGGCCTGACTTTAGCG

oTK090	rpnC_Do_fwd	CATCTTTTTCTCATGGCTCAAGAGCTAATC
oTK091	rpnC_Do_rev	ACTAACATGAGGTGGCAGCTCGTTCAGTAAAAGTATTG
oTK092	rpnC_UP_fwd	CATGGTAATTCACGACGAAAGTAAAACCGGGGGATAATC
oTK093	rpnC_UP_rev	GTTAATTACCGTGTGGCTTTGCGTGAAG
oTK094	rpnC_do_fwd	AGCCACACGGTAATTAACCTTATTTAAGTTACCTG
oTK095	rpnC_do_rev	ACTAACATGAGGTGGCGGAAGATCTGGCTCGTTATC
oTK096	rpnA_UP_fwd	CATGGTAATTCACGACGCTACCTGATTTTCGTCCTGC
oTK097	rpnA_UP_rev	GCCAACTCGTTGTTTGAATGCCGCGAC
oTK098	rpnA_DO_fwd	TCAAACAACGAGTTGGCTGCGGCGAGT
oTK099	rpnA_DO_rev	ACTAACATGAGGTGGCCCGAAGTGTGGCGTTGCATTG
oTK100	rpnB_UP_fwd	CATGGTAATTCACGACGCGATCAATCTGGTGCGCG
oTK101	rpnB_UP_rev	GGTGAAAGTAAATTTAAGATGACGCGACAATAACC
oTK102	rpnB-DO_fwd	ATCTTAAATTTACTTTCACCAGACGATCTTATC
oTK103	rpnB-DO_rev	ACTAACATGAGGTGGCACTCACGAGCAAAAAGAG
oTK112	pMS34-Nb.BsrDI up	ACACAGGAGGGACGTATGACAGAATATGACTTACATTTATATGCT
oTK113	pMS34-Nb.BsrDI do	CCAGCGGCCGCGTTTCCATCTTGCAATCTCTTCACT
oTK114	pMS34-*rhaBp-rpnC* (Δnt786-921) do	CCAGCGGCCGCGTTTGCGCTGCTCCCCTTTTATATATC
oTK121	R6K up ck	GCCACCTCATGTTAGTCATGCGC
oTK122	I-SceI do ck	CGTCGTGAATTACCATGTCAGCCG
oTK123	TN7L flanking primer	CATGGCAATTCTGGAAGAAATAG
oTK124	TN7R flanking primer	GATCTAAACTATGACAATAAAG
oTK125	NEW attTn7 DN	CATTAATAACGAAGAGATGAC
oTK126	NEW attTn7 UP	AATCTGTAACGTTCCGGGTTC
oTK127	pMS34-rpnA-mutD11A -up	ACACAGGAGGGACGTATGAGCAAAAAGCAGAGTTCCACCCCACACGCT GCGCTGTTC
oTK128	rpnA-mutD63A-up	GAAAGCTATTCCGCTGTGCTGTGGTCG
oTK129	rpnA-mutD63A-down	CGACCACAGCACAGCGGAATAGCTTTC
oTK130	rpnA-mutD82A-up	CTATTGTCTGATTGCACATCAAAGCACC
oTK131	rpnA-mutD82A-down	GGTGCTTTGATGTGCAATCAGACAATAG
oTK132	rpnA-mutQ84K-up	GTCTGATTGAACATAAAAGCACCTCAAAC
oTK133	rpnA-mutQ84K-down	GTTTGAGGTGCTTTTATGTTCAATCAGAC
oTK134	rpnA-mutQ84A-up	CTGATTGAACATGCAAGCACCTCAAAC
oTK135	rpnA-mutQ84A-down	GTTTGAGGTGCTTGCATGTTCAATCAG
oTK136	rpnA-mutR94A-up	CTGATCGCATTTGCCATGATGCGTTACG
oTK137	rpnA-mutR94A-up	CGTAACGCATCATGGCAAATGCGATCAG
oTK138	rpnA-mutD165A-up	GTCATGCCTGATGCTGAAATCATGCAG
oTK139	rpnA-mutD165A-down	CTGCATGATTTCAGCATCAGGCATGAC
oTK148	fhuA up ck	CGGGCCACGCATAATTTCAG

oTK149	fhuA do ck	TGAAAGCGAGCTTTTTGGCC
oTK161	pTXB1-rpnA-fwd	TTTAAGAAGGAGATATACATATGAGCAAAAAGCAGAGTTCCACC
oTK162	pTXB1-rpnA-rev	GTGCATCTCCCGTGATGCACTGACTCGCCGCAGCCAA
oTK163	pTXB1 fwd	CCGCGAAATTAATACGACTCA
oTK164	pTXB1 rev	GATTGCCATGCCGGTCAA
oTK176	ttcA_fwd	TTACCGCCGTAGAACATATTTAAG
oTK177	ttcA_rev (pKD4)	AGCCTACAATGCAAGAAAATCAACAAATTAC
oTK178	pdK4_fwd (ttcA)	TTCTTGCATTGTAGGCTGGAGCTGCTT
oTK179	pdK4_rev (ttcC)	CGCTTTTAACTGACATGGGAATTAGCCATG
oTK180	ttcC_fwd (pKD4)	CATGTCAGTTAAAAGCGACTCAATTCG
oTK181	ttcC_rev	CTGACGGTAATACCTTCAC

Table S2: *Enterobacteriacea panCD* segments analyzed to investigate *rpnC*/*yadD* distribution

Genome	GI number, GB number	length	coord start	coord end
E. aerogenes	991944407, CP014029.1	1316	3826998	3825683
C. koseri	673531252, LK931336.1	1328	3061505	3062832
C. rodentium ICC168	283783779, NC_013716.1	1379	172254	170876
K. oxytoca HKOP L1	612156087, CP004887.1	1329	5570706	5572034
K. oxytoca M1	662706586, CP008841.1	1329	2092600	2091272
S. enterica ssp indica	554684573, NZ_AOXI01000024.1	2470	79854	82323
S. enterica diarizonae	924626309, NZ_JZTQ01000011.1	2423	69072	71494
S. arizonae 62:z4,z23	161501984, NC_010067.1	1648	2733886	2735533
S. arizonae 62:Z36	686507741, CP006693.1	1648	2766877	2768524
Enterobacteriacea	440285501, NC_020063.1	2337	3876964	3879300
S. bongori NCTC 12419	339511397, FR877557.1	2471	191147	188677
S. bongori 48:z41	657146798, CP006692.1	2447	187771	185325
S. bongori N268-08	526225953, NC_021870.1	2471	188949	186479
C. freundii CAV1741	836563795, NZ_CP011657	1331	1967369	1968163
C. freundii FDA MicroDB61	835239805, NZ_JTBV01000001.1	1331	139357	140687
S. ent. typh FORC 15	818422575, CP011365.1	1330	224571	223242
S. ent. typh LT2	973795115, AE006468.2	1330	212360	211031
S. ent. paratyphi	56412276, NC_006511.1	1330	216586	215257
S. ent. choleraesuis	674188659, CP007639.1	1330	208434	207105
Escherichia sp KTE114	510899603, NZ_KE136626.1	2817	2039444	2036628
E. albertii KF1	569535620, CP007025.1	2619	1359282	1361900
Shigella sonnei 53G	377520096, NC_016822.1	2694	155934	153241
Shigella flexneri	828440138, CP007037.1	2482	146379	143898
E. coli K12 MG1655	556503834, NC_000913.3	2482	148795	146314
E. coli SQ2203	819394664, NZ_CP011324.1	2482	148795	146314
E. coli CHS199	852040470, NZ_KQ088929.1	2750	897255	894506
E. coli Nissle	660510169, CP007799.1	2799	156188	153390
E. coli 536	110640213, NC_008253.1	2792	154221	151430
E. coli APEC IM5155	742672810, CP005930.1	2472	3070880	3068409
Serratia proteamaculans	157368249, NC_009832.1	1458	4427337	4428794
Serratia fonticola	974016926, CP013913.1	1372	5556780	5558151
Pantoea sp	723239679, CP009866.1	1305	3042743	3044047

References

1. **Pan CQ, Lazarus RA.** 1999. Ca2+-dependent activity of human DNase I and its hyperactive variants. Protein Sci **8:**1780-1788.
2. **Merkel WK, Nichols BP.** 1996. Characterization and sequence of the Escherichia coli panBCD gene cluster. FEMS Microbiol Lett **143:**247-252.
3. **Steczkiewicz K, Muszewska A, Knizewski L, Rychlewski L, Ginalski K.** 2012. Sequence, structure and functional diversity of PD-(D/E)XK phosphodiesterase superfamily. Nucleic Acids Research **40:**7016-7045.
4. **Knizewski L, Kinch LN, Grishin NV, Rychlewski L, Ginalski K.** 2007. Realm of PD-(D/E)XK nuclease superfamily revisited: detection of novel families with modified transitive meta profile searches. BMC Struct Biol **7:**40.
5. Feb 2015. Database issue. The InterPro protein families database: the classification resource after 15 years. Nucleic Acids Res, 43.D213-221. http://eutils.ncbi.nlm.nih.gov/entrez/eutils/elink.fcgi?dbfrom=pubmed&id=25428371&retmode=ref&cmd=prlinks.
6. **Durfee T, Nelson R, Baldwin S, Plunkett G, Burland V, Mau B, Petrosino JF, Qin X, Muzny DM, Ayele M, Gibbs RA, Csörgo B, Pósfai G, Weinstock GM, Blattner FR.** 2008. The complete genome sequence of Escherichia coli DH10B: insights into the biology of a laboratory workhorse. J Bacteriol **190:**2597-2606.
7. **Studier F, Daegelen P, Lenski R, Maslov S, Kim J.** 2009. Understanding the Differences between Genome Sequences of Escherichia coli B Strains REL606 and BL21(DE3) and Comparison of the E. coli B and K-12 Genomes. Journal of Molecular Biology doi:10.1016/j.jmb.2009.09.021.
8. **Anonymous.** 2013. The NCBI Handbook [Internet]. *on* National Center for Biotechnology Information (US). http://www.ncbi.nlm.nih.gov/books/NBK143764/?report=classic. Accessed
9. May 2007. 3. Phylogenetic analysis of enteric species of the family Enterobacteriaceae using the oriC-locus. Syst Appl Microbiol, 30.180-188. http://eutils.ncbi.nlm.nih.gov/entrez/eutils/elink.fcgi?dbfrom=pubmed&id=16904857&retmode=ref&cmd=prlinks.
10. **Darling AC, Mau B, Blattner FR, Perna NT.** 2004. Mauve: multiple alignment of conserved genomic sequence with rearrangements. Genome Res **14:**1394-1403.
11. **Darling AE, Mau B, Perna NT.** 2010. progressiveMauve: multiple genome alignment with gene gain, loss and rearrangement. PLoS One **5:**e11147.
12. **Edgar RC.** 2004. MUSCLE: multiple sequence alignment with high accuracy and high throughput. Nucleic Acids Research **32:**1792-1797.
13. **Raleigh EA, Wilson G.** 1986. Escherichia coli K-12 restricts DNA containing 5-methylcytosine. Proc Natl Acad Sci U S A **83:**9070-9074.
14. **Kingston AW, Roussel-Rossin C, Dupont C, Raleigh EA.** 2015. Novel recA-Independent Horizontal Gene Transfer in Escherichia coli K-12. PLoS One **10:**e0130813.

15. **Piekarowicz A, Yuan R, Stein DC.** 1991. A new method for the rapid identification of genes encoding restriction and modification enzymes. Nucleic Acids Res **19:**1831-1835.
16. **Chong S, Mersha FB, Comb DG, Scott ME, Landry D, Vence LM, Perler FB, Benner J, Kucera RB, Hirvonen CA, Pelletier JJ, Paulus H, Xu MQ.** 1997. Single-column purification of free recombinant proteins using a self-cleavable affinity tag derived from a protein splicing element. Gene **192:**271-281.
17. **Baba T, Ara T, Hasegawa M, Takai Y, Okumura Y, Baba M, Datsenko KA, Tomita M, Wanner BL, Mori H.** 2006. Construction of Escherichia coli K-12 in-frame, single-gene knockout mutants: the Keio collection. Mol Syst Biol **2:**2006 0008.
18. **Sibley MH, Raleigh EA.** 2012. A versatile element for gene addition in bacterial chromosomes. Nucleic Acids Res **40:**e19.
19. **Datsenko KA, Wanner BL.** 2000. One-step inactivation of chromosomal genes in Escherichia coli K-12 using PCR products. Proc Natl Acad Sci U S A **97:**6640-6645.
20. **Tikh IB, Samuelson JC.** 2016. Leveraging modern DNA assembly techniques for rapid, markerless genome modification. Biology Methods and Protocols**:**(Accepted for Publication).
